# Pulse Wave Velocity, Mortality and Cardiovascular Disease in Chronic Kidney Disease: A Systematic Review and Meta‐Analysis

**DOI:** 10.1111/eci.70222

**Published:** 2026-05-13

**Authors:** Carlos Pascual‐Morena, Silvana Patiño‐Cardona, Miriam Garrido‐Miguel, Irene Martínez‐García, Maribel Lucerón‐Lucas‐Torres, Héctor Martínez‐Martínez, Elena Moreno‐Charco, José Alberto Martínez‐Hortelano

**Affiliations:** ^1^ Health and Social Research Center University of Castilla—La Mancha Cuenca Spain; ^2^ Faculty of Nursing University of Castilla—La Mancha Albacete Spain; ^3^ Age‐ABC Research Group, Health and Social Research Center University of Castilla—La Mancha Cuenca Spain; ^4^ Provincial Coordination and Inspection Service SESCAM Ciudad Real Spain; ^5^ Health, Gender, and Social Determinants Research Group, Health and Social Research Center University of Castilla—La Mancha Cuenca Spain

**Keywords:** end‐stage renal disease, epidemiology, Haemodialysis, prognosis, risk stratification, vascular ageing

## Abstract

**Background:**

Chronic kidney disease (CKD) is associated with high cardiovascular morbidity and mortality, which is mediated by arterial stiffness. Pulse wave velocity (PWV), as measured by the aortic (aPWV), brachial‐ankle (baPWV) and carotid‐radial (crPWV) indices or estimated by equations (ePWV), could be a powerful predictor of clinical events and mortality in this population. This study aimed to analyse the association between PWV and all‐cause mortality, cardiovascular mortality, and cardiovascular disease (CVD) risk in populations with CKD.

**Methods:**

A systematic search was conducted in PubMed, Scopus, and Web of Science from inception to January 2026. Observational studies estimating the association between PWV, categorically or by an increase of 1 m/s, and the outcomes of interest were included. Random‐effects meta‐analyses were performed, considering the type of PWV studied. The results are presented as hazard ratios (HR) and their 95% confidence intervals (95% CI).

**Results:**

Twenty‐eight studies were included in the systematic review, and 22 in the meta‐analyses. Considering a 1 m/s increase in aPWV, HRs of 1.16 (95% CI: 1.06, 1.28), 1.36 (95% CI: 1.15, 1.61), and 1.20 (95% CI: 1.02, 1.41) were found for all‐cause mortality, cardiovascular mortality, and CVD risk, respectively. The remaining associations for aPWV, baPWV, and ePWV were categorical and tended to be higher.

**Conclusions:**

Increased PWV was associated with an increased risk of mortality and CVD. However, given the low quality of the current evidence base, these findings should be interpreted with caution and more rigorous studies are needed to confirm their usefulness in clinical practice.

## Graphical abstract

This systematic review and meta‐analysis assessed the association between pulse wave velocity (PWV) and the mortality and morbidity in chronic kidney disease. An increase of every 1 m/s in aortic PWV was associated with a higher risk of all‐cause mortality (16%), cardiovascular mortality (36%), and cardiovascular disease (20%). These findings were consistent across aPWV, baPWV, and ePWV indices. Integrating PWV into clinical practise allows for more accurate cardiovascular risk stratification than traditional methods alone.

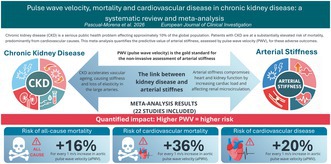

## 1 Introduction

Chronic kidney disease (CKD) is defined by the KDIGO guidelines as alterations to the structure or function of the kidneys that have been present for at least three months and directly affect health [1, 2]. Specifically, it is characterised by either a glomerular filtration rate (GFR) of less than 60 mL/min/1.73 m^2^ or the presence of markers of kidney damage, such as albuminuria, histological changes identified through a biopsy or structural abnormalities detected via imaging [3]. It is estimated to affect around 10% of the global population, making it a significant public health concern due to its progressive nature and the ageing population [4, 5]. The focus of clinical management is on therapies that delay complications associated with the disease itself and prevent progression to end‐stage renal disease (ESRD). It also involves continuous monitoring of patients and the detection of high‐risk individuals [6, 7, 8]. In fact, the main burden of the disease lies not only in kidney failure itself, but also in its systemic complications. Patients with CKD are therefore at high risk of cardiovascular morbidity and mortality, the latter being the leading cause of death even before progression to ESRD requiring dialysis [9, 10, 11, 12].

CKD, which is often associated with diabetes mellitus and hypertension, accelerates vascular ageing and promotes calcification of the tunica media (the middle layer of the arterial wall), resulting in arterial stiffness. This phenomenon is characterised by a loss of elasticity in large arteries and reduces vascular buffering capacity, thereby increasing ventricular afterload [13, 14]. Furthermore, the inability to dampen the pulse wave allows harmful pulsatile energy to be transmitted to the renal microcirculation. This causes capillary barotrauma, which accelerates the deterioration of glomerular filtration and increases the risk of cardiovascular mortality [15]. Although various other haemodynamic indicators exist, such as pulse pressure, the ankle‐brachial index and the augmentation index (Aix@75), pulse wave velocity (PWV) is considered the ‘gold standard’ for non‐invasively assessing arterial stiffness [16, 17]. PWV measurements can be taken using various regional approaches, such as carotid‐femoral (cfPWV), brachial‐ankle (baPWV) and carotid‐radial (crPWV), or by using estimated equations (ePWV). Determining their specific prognostic value in the renal population is crucial [16, 18].

Although previous meta‐analyses have evaluated the prognostic value of arterial stiffness in the general population and in conjunction with other markers of atherosclerosis in CKD [19, 20], the available evidence requires updating due to the recent publication of new cohorts. Furthermore, previous syntheses have been limited by the small number of studies focusing specifically on PWV [20]. Updating this review enables a more precise quantification of the relationship between various types of PWV and mortality and cardiovascular disease (CVD) in this population. Therefore, this systematic review and meta‐analysis aimed to summarise and estimate the association between arterial stiffness, as measured by PWV, and all‐cause mortality, cardiovascular mortality, and cardiovascular disease (CVD) in populations with CKD.

## 2 Methods

This systematic review and meta‐analysis was conducted in accordance with the Cochrane Collaboration Manual [21], the Preferred Reporting Items for Systematic Reviews and Meta‐Analyses (PRISMA), and the Meta‐Analysis of Observational Studies in Epidemiology (MOOSE) guidelines [22, 23]. It was also registered with the International Prospective Register of Systematic Reviews (PROSPERO) (CRD420261297193).

### 2.1 Search Strategy

A systematic search was conducted in Medline (via PubMed), Scopus and the Web of Science from their respective inception dates to January 2026. An open search was also performed using Google Scholar and grey literature sources, including OpenGrey, the Networked Digital Library of Theses and Dissertations, and the ProQuest database. The search used terms related to the study population (CKD), exposure (PWV measurement) and outcomes (all‐cause mortality, cardiovascular mortality and CVD), employing the Boolean operators ‘AND’ and ‘OR’. References included in previous reviews and in the studies included in this review were also examined. The search strategy is detailed in Appendix S1.

Systematic search was performed independently and manually by two authors (CP‐M and MG‐M), and disagreements were resolved by consensus or by a third author (JAM‐H).

### 2.2 Inclusion/Exclusion Criteria

Observational studies estimating the association between arterial stiffness, as measured by PWV, and the risk of all‐cause mortality, cardiovascular mortality, and CVD were included.

The inclusion criteria were as follows: (1) Population: individuals with CKD; (2) Exposure: arterial stiffness measured by PWV using validated devices (i.e., aPWV, cfPWV, baPWV, crPWV, or ePWV); (3) Comparator: comparison between different PWV percentiles or linearly by a 1 m/s increase; (4) Outcome: risk of all‐cause, cardiovascular mortality, and/or CVD; (5) Design: Observational studies. There were no language restrictions.

The exclusion criteria were as follows: (1) Studies have been conducted exclusively in kidney transplant recipients, given that this population has different clinical characteristics, cardiovascular risk factors and management strategies to those with CKD in stages 1–5, including the dialysis stage [3], (2) Abstracts, posters, commentaries or other documents with partial results; and (1) observational studies that did not report a risk estimate including a hazard ratio (HR), risk ratio (RR) or odds ratio (OR).

Study selection was performed independently and manually by two authors (CP‐M and MG‐M), and disagreements were resolved by consensus or by a third author (JAM‐H).

### 2.3 Data Extraction

The following information was extracted from the studies that were included in the systematic review: (1) author and year; (2) country in which the study was conducted; (3) study design and duration; (4) sample size, stages of CKD included, mean age, percentage of women, baseline HbA1c and estimated GFR (eGFR), and percentage of participants with diabetes mellitus; (5) type of PWV measurement (i.e., aPWV, cfPWV, baPWV, crPWV, or ePWV) and device used; (6) baseline PWV; (7) type of comparison (i.e., categorical according to percentiles or cut‐off points or linear according to a 1 m/s increase in PWV); and (8) outcomes (i.e., all‐cause mortality, cardiovascular mortality and/or CVD).

Data extraction was performed independently and manually by two authors (CP‐M and MG‐M), and disagreements were resolved by consensus or by a third author (JAM‐H).

### 2.4 Quality Assessment of Studies

The quality of the studies was assessed using the Study Quality Assessment Tool from the United States National Institute of Health (National Heart, Lung and Blood Institute) [24]. This tool comprises 14 items assessing various methodological, design, and statistical factors. Each study was classified as good if fewer than two items were rated as high risk, as fair if two items were rated as high risk, or as poor if more than two items were rated as high risk.

Quality assessment of studies was evaluated independently and manually by two authors (CP‐M and MG‐M), and disagreements were solved by consensus or by a third author (JAM‐H).

### 2.5 Grading the Quality of Evidence

The Grading of Recommendations, Assessment, Development and Evaluation (GRADE) tool was used to assess the quality and strength of the evidence [25]. The GRADE tool classifies the quality of evidence for the outcome from high to very low based on different domains, including the design of the included studies, risk of bias, imprecision, inconsistency, indirect evidence, evidence of publication bias, effect size, and possible unidentified confounding variables.

### 2.6 Statistical Analyses

An ad hoc table was created to summarise the results of the included studies, and a narrative synthesis of these was performed. The risk estimators used were those reported by the authors (i.e., HR, RR, or OR), along with their 95% confidence intervals (95% CI).

Meta‐analyses of the association between PWV and different outcomes were performed using the Hartung–Knapp–Sidik–Jonkman random‐effects model [26]. These estimates were obtained through meta‐analyses, which were categorised according to the type of PWV: aPWV, baPWV, and ePWV. cfPWV was considered a proxy for aPWV [27]. Furthermore, the data were separated according to the comparator; that is, whether the comparison was categorical (using cut‐off points or percentiles) or linear (using a 1 m/s increase in PWV). The original classification of HR, RR, or OR was maintained. Heterogeneity (*I* ^2^) was classified as not important if it was less than 30%, moderate if it was between 30% and 50%, substantial if it was between 50% and 75%, and considerable if it was greater than 75%. It was considered statistically significant when *p* < 0.05 [21, 28].

Secondary analyses were performed, including funnel plots and the Egger test, to determine whether there was evidence of publication bias. The Trim‐and‐Fill method was also used to estimate the possible effect of missing studies [29, 30]. Meta‐regressions were also performed according to age, the proportion of females, the proportion of participants with diabetes mellitus, and the length of the study. Finally, sensitivity analyses involving the exclusion of individual studies, as well as Galbraith plots, were performed to determine whether any particular study had a statistically significant influence on the overall result [31].

All statistical analyses were performed using Stata SE software (version 18; StataCorp, College Station, TX, USA).

## 3 Results

**FIGURE 1:**
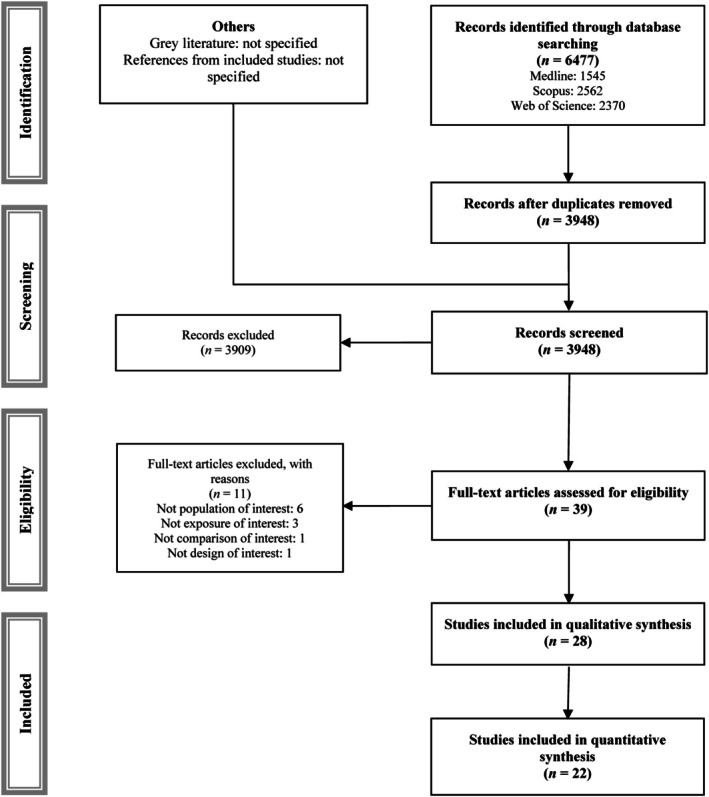
PRISMA flowchart of study selection.

Of the 6477 reports initially identified, 2529 duplicates were removed. The remaining studies were assessed based on their titles and abstracts, and 39 were read in full. Twenty‐eight of these studies were included in the systematic review [32, 33, 34, 35, 36, 37, 38, 39, 40, 41, 42, 43, 44, 45, 46, 47, 48, 49, 50, 51, 52, 53, 54, 55, 56, 57, 58, 59] and 22 were included in the meta‐analyses [32, 33, 34, 35, 36, 37, 40, 41, 42, 43, 44, 45, 47, 48, 49, 50, 51, 52, 53, 55, 56, 58] (Table 1, Figure 1), and 11 were excluded for justified reasons (Table S1) [60, 61, 62, 63, 64, 65, 66, 67, 68, 69, 70].

**TABLE 1:** Characteristics of the studies included in the systematic review.

References	Country	Design	Sample			Type of measure	Baseline PWV (m/s)	Comparison	Outcome		
			*N*	% Females	Age				AM	CM	CVD
Amemiya et al. (2011)	Japan	Retrospective	186	38.2	61.0 ± 12.0	baPWV	18.7 ± 5.4	Tertiles	✓	—	—
Avramovski et al. (2014)	Macedonia	Prospective	80	33.8	59.3 ± 11.8	cfPWV	12.5 ± 2.0	> 11.8 vs. < 11.8 m/s	✓	✓	—
								Linear			
Bao et al. (2019)	China	Prospective	181	NA	NA	cfPWV, crPWV and PWV ratio	NA	Linear	✓	✓	—
Baumann et al. (2014)	Germany	Prospective	129	54.0	59.2 ± 15.1	aPWV	10.5 ± 3.0	> 10.0 vs. < 10.0 m/s	✓	—	—
								Linear			
Blacher et al. (2003)	France	Prospective	242	39.0	52.0 ± 16.0	cfPWV	11.1 ± 3.1	Linear	✓	✓	—
Cui et al. (2024)	China	Retrospective	1173	27.1	NA	ePWV	10.4 ± 2.3	Quartiles	✓	—	—
								Linear			
Eun Yoon et al. (2013)	South Korea	Retrospective	241	45.6	52.9 ± 9.2	baPWV	14.6 ± 2.6	Linear	—	—	✓
Feng et al. (2025)	US	Prospective	4669	51.9	71.9	ePWV	11.5	Linear	✓	✓	
Ferreira et al. (2017)	France	Prospective	278	38.8	53.0 ± 16.0	aPWV	11.3 ± 3.0	> 12.0 vs. < 12.0 m/s	✓	✓	—
								Linear			
Fortier et al. (2015)	Canada	Prospective	310	40.0	67.0	cfPWV	13.5 ± 4.1	Tertiles	✓	—	—
						crPWV	8.8 ± 1.7	Linear			
						PWV ratio	1.6 ± 0.5				
Han et al. (2016)	China	Prospective	1499	58.0	61.4	cfPWV	12.3 ± 3.3	Linear	—	—	✓
Kato et al. (2010)	Japan	Prospective	194	34.5	64.0 ± 12.0	baPWV	16.7 ± 5.1	Tertiles	✓	—	✓
Kato A et al. (2012)	Japan	Prospective	135	32.6	60.0 ± 11.0	baPWV	16.0 ± 3.6	Tertiles	✓	✓	✓
Kitahara et al. (2005)	Japan	Prospective	785	35.5	60.2 ± 12.5	baPWV	21.4 ± 6.8	Quartiles	✓	—	—
Kuwuhara et al. (2013)	Japan	Prospective	300	39.0	61.0 ± 9.6	baPWV	18.7 ± 4.9	Linear	—	✓	—
Matschkal et al. (2019)	Germany	Prospective	344	32.0	69.3	aPWV	10.0	Linear	✓	✓	—
Ng et al. (2023)	Taiwan	Prospective	130	47.7	63.2 ± 12.9	cfPWV	10.4 ± 3.3	Linear	✓	✓	—
Otsuka et al. (2019)	Japan	Prospective	104	37.0	71.0 ± 7.0	baPWV	22.6 ± 7.5	Tertiles	—	—	✓
Shin et al. (2009)	South Korea	Prospective	72	41.7	50.4 ± 13.0	aPWV	8.1 ± 1.1	Linear	—	—	✓
Tanaka et al. (2011)	Japan	Prospective	445	40.7	63.0 ± 11.0	baPWV	NA	Tertiles	✓	✓	✓
Tripepi et al. (2018)	France	Prospective	533	39.2	58.5 ± 16.7	aPWV	11.7	Linear	✓	—	✓
Townsend et al. (2018)	US	Prospective	2795	43.6	59.9 ± 10.8	cfPWV	NA	Tertiles	✓	—	—
Verbeke et al. (2011)	Europe	Prospective	1076	NA	NA	cfPWV	NA	Tertiles	✓	—	—
								Linear			
Wang et al. (2018)	China	Retrospective	254	38.6	61.4 ± 15.3	baPWV	17.6 ± 5.8	Tertiles	✓	—	—
								Linear			
Wang et al. (2026)	China	Retrospective	233	50.2	57.2 ± 14.5	cfPWV	12.8 ± 3.6	Linear	✓	✓	—
						crPWV	9.9 ± 2.0				
						cdPWV	9.4 ± 3.7				
Xu et al. (2015)	China	Prospective	59	47.5	52.80	cfPWV	NA	> 12.0 vs. < 12.0 m/s	—	—	✓
Zang et al. (2026)	US	Prospective	3908	50.8	NA	ePWV	9.9 ± 2.4	Quartiles	✓	✓	—
Zoungas et al. (2007)	Australia	Prospective	315	32.4	55.0 ± 13.0	afPWV	NA	Tertiles	—	—	✓
	New Zealand							Linear			

Abbreviations: AM, All‐cause mortality; aPWV, aortic pulse wave velocity; baPWV, brachial‐ankle pulse wave velocity; cdPWV, carotid‐dorsalis pedis pulse wave velocity; cfPWV, carotid‐femoral pulse wave velocity; CM, cardiovascular mortality; crPWV, carotid‐radial pulse wave velocity; CVD, cardiovascular disease; ePWV, estimated pulse wave velocity; m/s, meters per second; NA, not available; US, United States.

The studies included in the systematic review and meta‐analysis were conducted in various countries across Asia, Europe, and North America. Of these, 23 were prospective and 5 were retrospective. The sample sizes ranged from 59 to 4669 participants, with mean ages ranging from 50.4 to 71.9 years, and percentages of women ranging from 27.1% to 58.0%. Most studies used cfPWV or baPWV to measure arterial stiffness, though some used ePWV or crPWV. Twenty‐one studies estimated the risk of all‐cause mortality, 12 estimated the risk of cardiovascular mortality, and 10 estimated the risk of CVD. Regarding additional baseline characteristics, most studies included populations with advanced CKD and a high prevalence of diabetes mellitus. They employed models adjusted for multiple clinical covariates and various measurement devices validated over heterogeneous follow‐up periods. The characteristics of the observational studies included in the systematic review are described in Tables 1 and S2.

### 3.1 Finding of the Systematic Review

Table 2 summarises the associations between PWV and different reported outcomes in the included studies.

**TABLE 2:** Results of the systematic review.

References	Type of PWV	Estimator	Comparison	All‐cause mortality (95% CI)	Cardiovascular mortality (95% CI)	Cardiovascular disease (95% CI)
Amemiya et al. (2011)	baPWV	HR	T3 vs. T1	2.06 (0.52, 8.11)	—	—
Avramovski et al. (2014)	cfPWV	HR	> 11.8 vs. < 11.8 m/s	4.29 (1.61, 11.51)^b^	4.36 (2.01, 102.77)^b^	—
Avramovski et al. (2014)	cfPWV	HR	Linear	1.29 (1.09, 1.54)	1.43 (1.17, 1.75)	—
Bao et al. (2019)	cfPWV	HR	Linear	1.27 (1.16, 1.38)^b^	—	—
Bao et al. (2019)	PWV ratio	HR	Linear	2.42 (1.80, 3.25)^b^	2.08 (1.16, 3.71)	—
Baumann et al. (2014)	aPWV	HR	> 10.0 vs. < 10.0 m/s	5.1 (1.1, 22.9)	—	—
Baumann et al. (2014)	aPWV	OR	Linear	1.26 (1.01, 1.57)	—	—
Blacher et al. (2003)	cfPWV	HR	Linear	1.14 (1.05, 1.24)	1.14 (1.03, 1.26)	
Cui et al. (2024)—1	ePWV	HR	Q3 vs. Q2	1.17 (0.70, 1.96)	—	—
Cui et al. (2024)—2	ePWV	HR	Q4 vs. Q2	2.04 (1.31, 3.19)	—	—
Eun Yoon et al. (2013)	baPWV	OR	Linear	—	—	1.22 (1.00, 1.49)
Feng et al. (2025)	ePWV	HR	Linear	1.28 (1.24, 1.32)	1.28 (1.21, 1.36)	—
Ferreira et al. (2017)	aPWV	HR	> 12 vs. < 12 m/s	54.51 (6.46, 459.00)	97.62 (7.17, 1329.00)	—
Ferreira et al. (2017)	aPWV	HR	Linear	1.84 (1.31, 2.58)	2.03 (1.35, 3.04)	—
Fortier et al. (2015)	cfPWV	HR	Linear	1.06 (1.03, 1.10)^b^	—	—
Fortier et al. (2015)	crPWV	HR	Linear	0.88 (0.79, 0.97)^b^	—	—
Fortier et al. (2015)	PWV ratio	HR	T3 vs. T1	1.25 (1.03, 1.51)	—	—
Han et al. (2016)	cfPWV	HR	Linear	—	—	1.30 (1.02, 1.64)
Kato A et al. (2010)	baPWV	HR	T3 vs. T1	1.33 (0.51, 3.10)	—	1.60 (0.70, 3.66)
Kato et al. (2012)	baPWV	HR	T3 vs. T1	1.70 (0.60, 4.70)	16.9 (1.10, 251.80)	3.40 (0.70, 16.20)
Kitahara et al. (2005)	baPWV	HR	Q4 vs. Q1	1.79 (0.93, 3.48)	—	—
Kuwahara et al. (2013)	baPWV	HR	Linear	—	1.06 (1.02, 1.11)^b^	1.05 (1.01, 1.09)^b^
Matschkal et al. (2019)	aPWV	HR	Linear	1.44 (0.82, 2.53)	1.35 (1.13, 1.61)^b^	—
Ng et al. (2023)	cfPWV	HR	Linear	1.17 (1.03, 1.33)	1.32 (1.13, 1.53)	—
Otsuka et al. (2019)	baPWV	HR	T3 vs. T1	—	—	1.19 (0.64, 2.20)^b^
Shin et al. (2009)	aPWV	HR	Linear	—	—	1.63 (1.09, 2.44)
Tanaka et al. (2011)—1	baPWV	HR	T2 vs. T1	2.02 (0.69, 5.91)	3.22 (0.83, 12.44)	1.16 (0.62, 1.74)
Tanaka et al. (2011)—2	baPWV	HR	T3 vs. T1	2.63 (0.89, 7.80)	3.61 (0.97, 13.45)	1.51 (0.68, 1.97)
Tripepi et al. (2018)—1	aPWV	HR	Linear	1.10 (1.02, 1.20)	—	1.17 (1.07, 1.28)
Tripepi et al. (2018)—2	aPWV	HR	Linear	1.04 (0.97, 1.10)	—	1.06 (0.98, 1.14)
Townsend et al. (2018)—1	cfPWV	HR	T2 vs. T1	1.41 (1.02, 1.95)	—	—
Townsend et al. (2018)—2	cfPWV	HR	T3 vs. T1	1.72 (1.24, 2.38)	—	—
Verbeke et al. (2011)	cfPWV	HR	T2 vs. T1	1.28 (0.91, 1.81)^a^	—	—
Verbeke et al. (2011)	cfPWV	HR	T3 vs. T1	1.94 (1.38, 2.73)^a^	—	—
Verbeke et al. (2011)	cfPWV	HR	Linear	1.15 (1.09, 1.23)^a^	—	—
Wang et al. (2018)	baPWV	HR	T3 vs. T1	2.97 (1.23, 7.16)	—	—
Wang et al. (2018)	baPWV	HR	Linear	1.07 (1.03, 1.12)	—	—
Wang et al. (2026)	cfPWV	HR	Linear	1.04 (0.97, 1.11)	1.15 (1.05, 1.26)	—
Wang et al. (2026)	crPWV	HR	Linear	0.94 (0.85, 1.04)	0.97 (0.85, 1.12)	—
Wang et al. (2026)	cdPWV	HR	Linear	1.04 (0.97, 1.11)	1.05 (0.98, 1.13)	—
Xu et al. (2015)	cfPWV	HR	> 12.0 vs. < 12.0 m/s	—	—	1.48 (1.01, 2.18)
Zang et al. (2026)—1	ePWV	HR	Q3 vs. Q1	2.53 (1.55, 4.15)	1.52 (0.70, 3.32)	—
Zang et al. (2026)—2	ePWV	HR	Q4 vs. Q1	4.12 (2.38, 7.13)	2.71 (1.24, 5.95)	—
Zoungas et al. (2007)	afPWV	HR	T3 vs. T1	—	—	2.49 (1.01, 6.14)
Zoungas et al. (2007)	afPWV	HR	Linear	—	—	1.14 (1.07, 1.26)

Abbreviations: afPWV, artery‐femoral pulse wave velocity; aPWV, aortic pulse wave velocity; baPWV, brachial‐ankle pulse wave velocity; cdPWV, carotid‐dorsalis pedis pulse wave velocity; cfPWV, carotid‐femoral pulse wave velocity; crPWV, carotid‐radial pulse wave velocity; ePWV, estimated pulse wave velocity; HR, hazard ratio; m/s, meters per second; OR, odds ratio; PWV ratio, pulse wave velocity ratio; Q1, first quartile; Q2, second quartile; Q3, third quartile; Q4, fourth quartile; T1, first tertile; T2, second tertile; T3, third tertile.

^a^ All‐cause mortality + first CVD event, therefore not included in the meta‐analysis.

^b^ Not adjusted.

Categorical comparisons based on tertiles, quartiles or predefined PWV cut‐off points revealed an association with all‐cause mortality risk, with statistically significant HRs ranging from 1.25 to 54.51. While some studies did not reach statistical significance, all showed a trend towards an increased risk. These findings were similar to those for the association between categorised PWV and the risk of cardiovascular mortality and CVD.

In linear comparisons, i.e., the change in risk for each 1 m/s increase in PWV, most studies showed a positive trend towards an increased risk of all‐cause mortality, with HR estimates mostly falling within the range of 1.04–1.84. This range was statistically significant in more than half of the studies. Regarding cardiovascular mortality, a general trend towards increased risk was also observed, with linear HRs ranging from 0.97 to 2.08. Finally, with regard to CVD risk, increased PWV was consistently associated with a higher risk, showing statistically significant linear HRs ranging from 1.05 to 1.63 and ORs reaching 1.22 per unit increase.

### 3.2 Quality Assessment of Studies

According to the Study Quality Assessment Tools, 25 out of 28 (89.3%) of the included studies were rated as ‘good’, 2 out of 28 (7.1%) were rated as ‘fair’, and 1 out of 28 (3.6%) was rated as ‘poor’. The most commonly affected domains were the lack of justification for the sample size and the lack of blinding of outcome assessors to participants' exposure status. The risk of bias assessment is described in Supplementary Table S3.

### 3.3 Grading the Quality of Evidence

According to the GRADE tool, the quality of the evidence was rated as low for the associations between baPWV (categorical) and all‐cause mortality, ePWV (categorical) and cardiovascular mortality, and baPWV (linear) and CVD. For the remaining outcomes, the rating was very low. The assessment of the quality of the evidence is described in Table S4.

### 3.4 Meta‐Analyses

**FIGURE 2:**
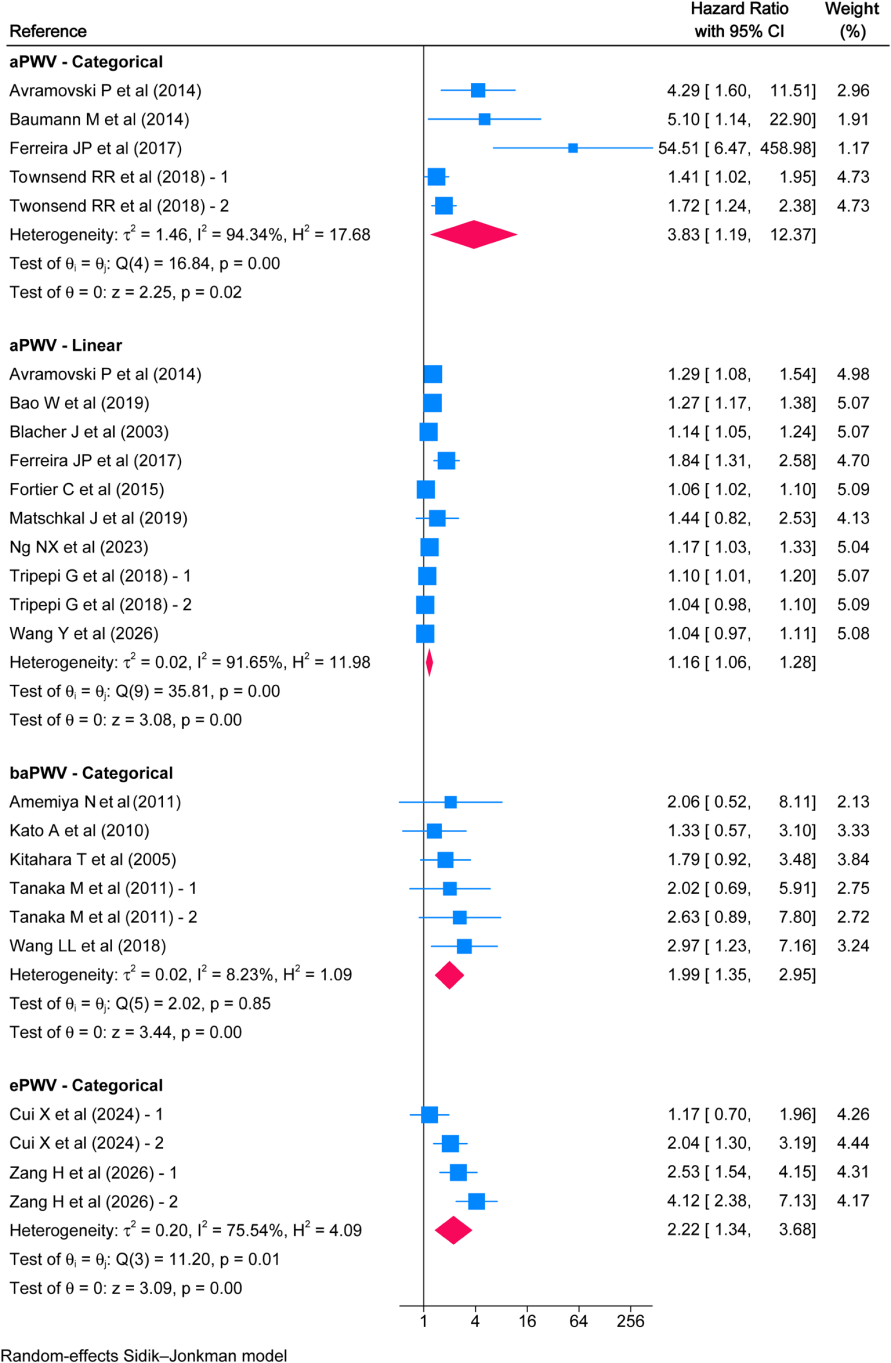
Association between pulse wave velocity and all‐cause mortality in a population with chronic kidney disease.

**FIGURE 3:**
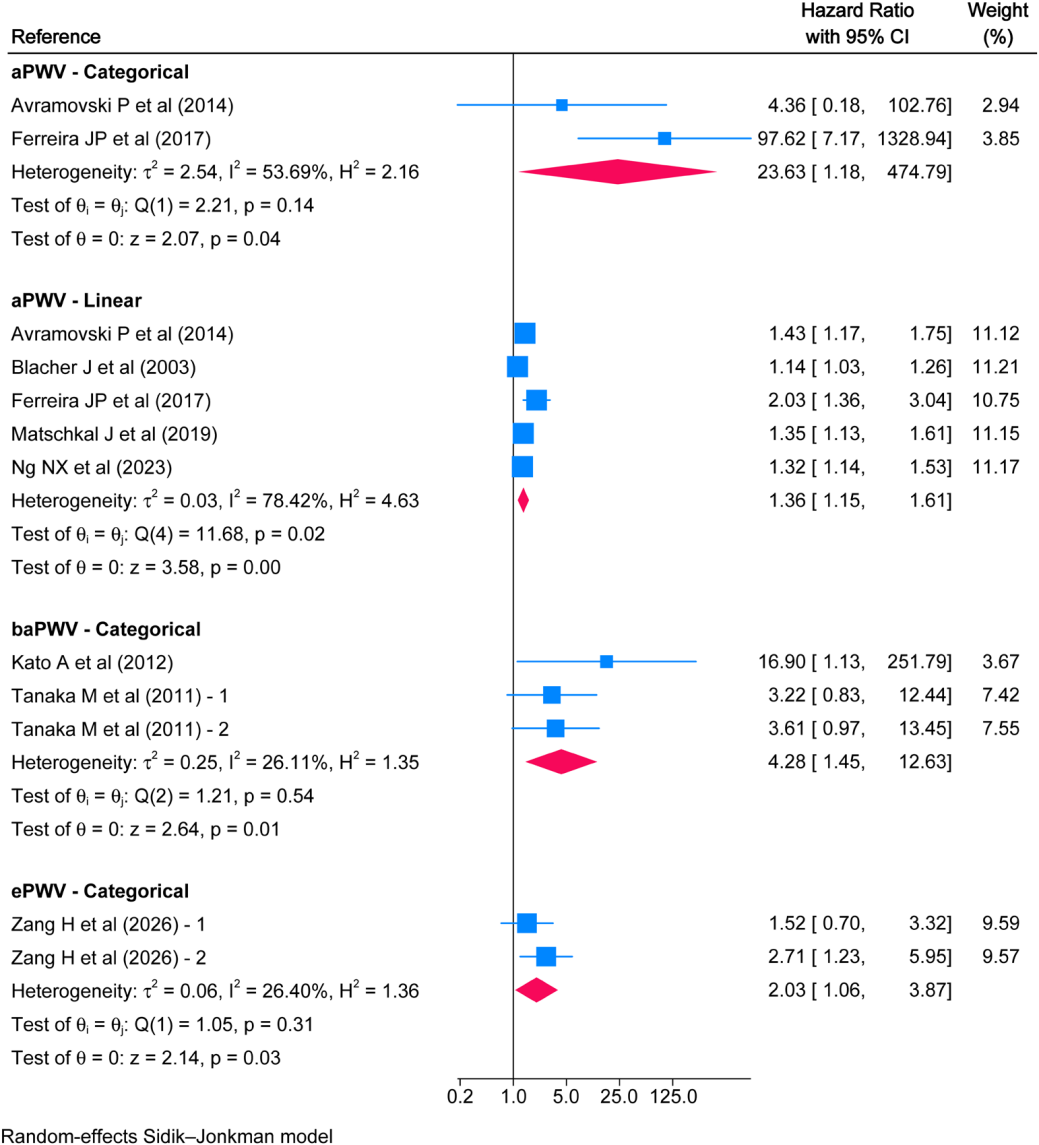
Association between pulse wave velocity and cardiovascular mortality in a population with chronic kidney disease.

**FIGURE 4:**
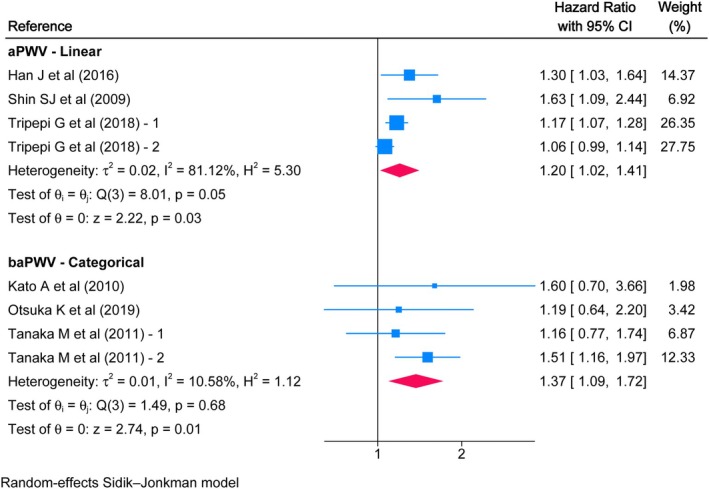
Association between pulse wave velocity and risk of cardiovascular disease in a population with chronic kidney disease.

High aPWV values were associated with an increased risk of all‐cause mortality, with an HR of 3.83 (95% CI: 1.19, 12.37) compared to low values. For baPWV, the HR was 1.99 (95% CI: 1.35, 2.95), and for ePWV, the HR was 2.22 (95% CI: 1.34, 3.68). Furthermore, for every 1 m/s increase in aPWV, the risk of all‐cause mortality was HR 1.16 (95% CI: 1.06, 1.28) (Figure 2). Moreover, high aPWV values were associated with a higher risk of cardiovascular mortality with an HR of 23.63 (95% CI: 1.18, 474.79) and a linear increase in risk with an HR of 1.36 (95% CI: 1.15, 1.61) for every additional m/s. Similarly, an HR of 4.28 (95% CI: 1.45, 12.63) was estimated for high baPWV values, while for ePWV, the HR was 2.03 (95% CI: 1.06, 3.87) (Figure 3). Finally, the linear association of aPWV with the risk of CVD showed an HR of 1.20 (95% CI: 1.02, 1.41), while elevated baPWV values were associated with an HR of 1.37 (95% CI: 1.09, 1.72) (Figure 4). Overall, there was moderate to considerable heterogeneity, except for categorical baPWV, which was not important in any of the three outcomes.

### 3.5 Secondary Analyses

Evidence of publication bias was found for the categorical and linear association of aPWV with all‐cause mortality (*p* = 0.003 and *p* = 0.007, respectively), as well as for the linear association of aPWV with cardiovascular mortality (*p* = 0.001) and CVD (*p* = 0.025) (Figures S1–S3, Table S5). Further analysis using the trim‐and‐fill method revealed a slight trend towards lower estimates after missing studies were imputed, though the results remained consistent (Table S5). Meta‐regression analysis revealed that participant age was a significant covariate in the relationship between categorical aPWV and all‐cause mortality (*p* = 0.003). However, no other statistically significant associations were identified (Table S6). Sensitivity analyses showed that no individual study significantly altered the overall estimates (Figures S4–S6). Regarding the Galbraith charts, Ferreira et al. [40] study on all‐cause mortality (linear aPWV) fell outside the expected 95% CI, identifying it as an outlier (Figures S7–S9).

## 4 Discussion

### 4.1 Main Findings

Our results showed that PWV was strongly associated with an increased risk of all‐cause mortality, cardiovascular mortality, and CVD, regardless of the type of PWV used. Specifically, a significant increase in risk was observed when comparing high and low PWV levels, as well as for each 1 m/s increase in the measurement. This suggests a consistent relationship in this high‐risk population. Finally, heterogeneity was generally moderate to considerable, except for categorical low PWV, for which it was not significant. However, one study was an outlier, and trim‐and‐fill analysis slightly reduced the effect size of some associations.

### 4.2 Interpretation

Our analysis showed that there was a 16% increased risk of all‐cause mortality for every 1 m/s increase in aPWV. The effect size was larger in categorical analyses when comparing more extreme phenotypes. However, there was high heterogeneity, particularly regarding aPWV. The main outlier was Ferreira et al., whose high HRs originated from a haemodialysis cohort in which arterial stiffness indicates premature and lethal vascular ageing, particularly in patients under 60 years of age. Furthermore, the results were imprecise due to the small number of events in participants with lower arterial stiffness [40]. Regarding ePWV, Zang et al. reported a higher risk than Cui et al. This discrepancy reflects baseline differences. Zang et al. evaluated the general outpatient population, whereas Cui analysed critically ill patients in the intensive care units. The latter group has a high baseline mortality rate due to severe comorbidities, which tends to attenuate the relative magnitude of the association [37, 58].

Regarding cardiovascular morbidity and mortality, our results indicate a 36% increase in mortality and a 20% increase in the risk of CVD for every 1 m/s increase in aPWV. These associations are magnified in categorical comparisons when phenotypes of extreme stiffness are considered. Ferreira et al. report very high risks in haemodialysis patients, specifically those under 60 years of age, where arterial stiffness denotes accelerated vascular ageing and severe, irreversible calcification, which largely explains the marked heterogeneity in cardiovascular mortality [40]. Meanwhile, the greater magnitude of association with CVD risk reported by Shin et al. could be attributed to the distribution of events in their cohort, which was mainly composed of cerebral infarctions rather than cardiac events [50]. This therefore supports the hypothesis that aortic stiffness enables damaging pulsatile energy to be transmitted to the cerebral microcirculation (barotrauma), causing cerebrovascular damage [71].

The relationship between arterial stiffness and renal function is not just an epidemiological association; it is also a complex, bidirectional pathophysiological interaction [72]. It is well known that CKD promotes arterial hardening through vascular calcification, bone mineral disorders, the accumulation of advanced glycation end products and oxidative stress [11], there is substantial evidence that arterial stiffness itself contributes to the progression of kidney disease and target organ damage [73]. From a pathophysiological perspective, the kidneys are low‐resistance, high‐flow organs that depend on the elastic buffering capacity of the large arteries to safeguard the microcirculation against pulsatile pressure fluctuations. When the aorta stiffens and loses this capacity, excessive pulsatile energy is transmitted directly to the renal vascular beds. This leads to glomerular hypertension, hyperfiltration and, ultimately, glomerulosclerosis and nephron loss [72, 73].

It is worth noting that although studies of kidney transplant recipients were excluded due to differences in cardiovascular and pharmacological dynamics, our results are consistent with previous findings. Participants in this group have been shown to exhibit significantly lower arterial stiffness than subjects with native CKD matched for renal function (10.1 vs. 11.0 m/s). This appears to be linked to reduced levels of protein‐bound uremic toxins, specifically indoxyl sulphate, after renal function has recovered. Despite this lower magnitude of stiffness, PWV remains an independent risk factor for overall mortality in this population (HR = 1.29). Notably, however, aortic stiffness is not statistically associated with microvascular graft damage (Banff CV score), which indicates an efficient myogenic response and haemodynamic autoregulation in response to barotraumatic stress from pulsatile energy [74, 75]. Therefore, integrating this perspective reinforces the usefulness of PWV as a cross‐cutting prognostic biomarker across the entire pathophysiological spectrum of kidney disease.

From a cardiovascular perspective, an increase in PWV alters ventriculo‐arterial coupling, generating adverse haemodynamic consequences that explain the high mortality risk observed in our meta‐analysis [76]. The early return of reflected waves during systole increases central aortic pressure and left ventricular afterload. This promotes left ventricular hypertrophy and increases the heart muscle's demand for oxygen [77]. This phenomenon simultaneously reduces aortic diastolic pressure and compromises coronary artery perfusion, predominantly during diastole. This creates an imbalance between oxygen supply and demand, predisposing the heart to ischaemia, heart failure, and fatal arrhythmias independently of traditional risk factors [76, 77].

Our findings have substantial clinical implications, given that ESRD has an alarmingly high annual mortality rate of approximately 15%, which is even higher in the first year after starting dialysis [78, 79]. In this context of high baseline risk, the 16% increase in risk for each m/s of aPWV that we report is significant; mathematically, this implies that annual absolute mortality could increase by approximately three additional deaths per 100 dialysis patients for each unit increase in arterial stiffness. Therefore, routinely implementing PWV would enable these patients to be re‐stratified beyond traditional risk factors, identifying a ‘very high‐risk’ subgroup that would benefit from more aggressive cardioprotective strategies and intensive cardiovascular monitoring to mitigate the burden of excess mortality.

### 4.3 Limitations

This study has several limitations that should be considered. First, some meta‐analyses included a limited number of studies, meaning that secondary analyses such as the assessment of publication bias or meta‐regressions were exploratory in nature due to low statistical power [21]. Second, the quality of the evidence was assessed as low to very low, precluding direct causal inference. Future studies are needed not only to address the existing limitations, but also to evaluate whether reducing arterial stiffness reduces mortality and CVD in CKD patients. Third, while all studies detailed the use of PWV measurement tools, using different tools could impact the estimates obtained. Fourth, while most studies adjusted the results for various covariates, using different models could slightly alter the estimates obtained.

## 5 Conclusions

Our study suggests that arterial stiffness, as measured by PWV, is an independent predictor of all‐cause and cardiovascular mortality, as well as CVD, in patients with CKDCKD. This association remains consistent regardless of the measurement method used (aPWV, baPWV, or ePWV), with a significant increase in risk observed for each additional m/s. Given the high baseline mortality in this population, integrating PWV into clinical practise could improve the stratification of cardiovascular risk beyond traditional factors, enabling the identification of vulnerable phenotypes. Further studies are needed to determine whether reducing arterial stiffness through therapeutic interventions can reverse this adverse prognosis.

## Author Contributions

**Carlos Pascual‐Morena** and **Miriam Garrido‐Miguel:** conceptualisation. **Carlos Pascual‐Morena** and **José Alberto Martínez‐Hortelano:** methodology. **Carlos Pascual‐Morena** and **Miriam Garrido‐Miguel:** data curation and investigation. **Carlos Pascual‐Morena**, **Silvana Patiño‐Cardona**, **Irene Martínez‐García**, **Maribel Lucerón‐Lucas‐Torres** and **Elena Moreno‐Charco:** formal analysis. **Carlos Pascual‐Morena**, **Silvana Patiño‐Cardona**, **Héctor Martínez‐Martínez** and **Elena Moreno‐Charco:** validation and visualisation. **Carlos Pascual‐Morena**, **Miriam Garrido‐Miguel** and **José Alberto Martínez‐Hortelano:** writing – original draft preparation. **Carlos Pascual‐Morena**, **Silvana Patiño‐Cardona**, **Miriam Garrido‐Miguel**, **Irene Martínez‐García**, **Maribel Lucerón‐Lucas‐Torres**, **Héctor Martínez‐Martínez**, **Elena Moreno‐Charco**, **José Alberto Martínez‐Hortelano:** writing – review and editing. **Carlos Pascual‐Morena:** supervision. **Carlos Pascual‐Morena:** funding acquisition. **Carlos Pascual‐Morena:** project administration. All authors have read and agreed to the published version of the manuscript.

## Funding

This study was supported by the University of Castilla‐La Mancha, of which the authors are members.

## Ethics Statement

The authors have nothing to report.

## Conflicts of Interest

The authors declare no conflicts of interest.

## Supporting information


**Table S1:** Studies excluded with justified reasons.
**Table S2:** Additional baseline characteristics of the included studies and their participants.
**Table S3:** Quality assessment of cohorts and cross‐sectional studies.
**Table S4:** Quality of evidence assessment.
**Table S5:** Publication bias and Trim‐and‐Fill test.
**Table S6:** Metaregressions analyses.
**Figure S1:** Publication bias assessment for the risk of all‐cause mortality.
**Figure S2:** Publication bias assessment for the risk of cardiovascular mortality.
**Figure S3:** Publication bias assessment for the risk of cardiovascular disease.
**Figure S4:** Sensitivity analyses for the risk of all‐cause mortality.
**Figure S5:** Sensitivity analyses for the risk of cardiovascular mortality.
**Figure S6:** Sensitivity analyses for the risk of cardiovascular disease.
**Figure S7:** Galbraith plots for the risk of all‐cause mortality.
**Figure S8:** Galbraith plots for the risk of cardiovascular mortality.
**Figure S9:** Galbraith plots for the risk of cardiovascular disease.
**Appendix S1:** Search strategy.

## Data Availability

The data that support the findings of this study are available from the corresponding author upon reasonable request.
