# Supplementary material for: Pulse Wave Velocity, Mortality and Cardiovascular Disease in Chronic Kidney Disease: A Systematic Review and Meta‐Analysis
Source: Eur J Clin Invest. 2026 May 13;56:e70222. doi: 10.1111/eci.70222 (PMC13170642; doi:10.1111/eci.70222)
Supplement: Supplementary file 1 — Table S1: Studies excluded with justified reasons. Table S2: Additional baseline characteristics of the included studies and their participants. Table S3: Quality assessment of cohorts and cross‐sectional studies. Table S4: Quality of evidence assessment. Table S5: Publication bias and Trim‐and‐Fill test. Table S6: Metaregressions analyses. Figure S1: Publication bias assessment for the risk of all‐cause mortality. Figure S2: Publication bias assessment for the risk of cardiovascular mortality. Figure S3: Publication bias assessment for the risk of cardiovascular disease. Figure S4: Sensitivity analyses for the risk of all‐cause mortality. Figure S5: Sensitivity analyses for the risk of cardiovascular mortality. Figure S6: Sensitivity analyses for the risk of cardiovascular disease. Figure S7: Galbraith plots for the risk of all‐cause mortality. Figure S8: Galbraith plots for the risk of cardiovascular mortality. Figure S9: Galbraith plots for the risk of cardiovascular disease. Appendix S1: Search strategy. [file ECI-56-e70222-s001.docx]

**SUPPLEMENTARY MATERIAL**

**Pulse wave velocity, mortality and cardiovascular disease in chronic kidney disease: a systematic review and meta-analysis**

**INDEX**

**Table S1.** Studies excluded with justified reasons.

**Table S2**. Additional baseline characteristics of the included studies and their participants.

**Table S3.** Quality assessment of cohorts and cross-sectional studies.

**Table S4.** Quality of evidence assessment.

**Table S5.** Publication bias and Trim-and-Fill test.

**Table S6.** Metaregressions analyses.

**Figure S1.** Publication bias assessment for the risk of all-cause mortality.

**Figure S2.** Publication bias assessment for the risk of cardiovascular mortality.

**Figure S3.** Publication bias assessment for the risk of cardiovascular disease.

**Figure S4.** Sensitivity analyses for the risk of all-cause mortality.

**Figure S5.** Sensitivity analyses for the risk of cardiovascular mortality.

**Figure S6.** Sensitivity analyses for the risk of cardiovascular disease.

**Figure S7.** Galbraith plots for the risk of all-cause mortality.

**Figure S8.** Galbraith plots for the risk of cardiovascular mortality.

**Figure S9.** Galbraith plots for the risk of cardiovascular disease.

**Appendix S1.** Search strategy.

**Table S1.** Studies excluded with justified reasons.

| **Reference** | **Main reason** |
| --- | --- |
| Chen T et al (2025)^1^ | Not comparison of interest |
| Choi SW et al (2010)^2^ | Not population of interest |
| Cote N et al (2024)^3^ | Not exposure of interest |
| Guerin AP et al (2001)^4^ | Not design of interest |
| Heleniak Z et al (2022)^5^ | Not population of interest |
| Korogiannou M et al (2019)^6^ | Not population of interest |
| Melilli E et al (2015)^7^ | Not population of interest |
| Moissl-Blanke AP et al (2025)^8^ | Not exposure of interest |
| Shi J et al (2025)^9^ | Not population of interest |
| Shoji T et al (2010)^10^ | Not exposure of interest |
| Verbeke F et al (2011)^11^ | Not population of interest |

Supplementary references

1. Chen T, Yin H, Zhou Y, Liang M. Relationship between estimated pulse wave velocity trajectories and cardiovascular disease risk in patients with cardiovascular-kidney-metabolic syndrome stages 0-3. *Nutr Metab Cardiovasc Dis*. 2025;35(11):104192. doi:10.1016/j.numecd.2025.104192

2. Choi SW, Yun WJ, Kim HY, et al. Association between albuminuria, carotid atherosclerosis, arterial stiffness, and peripheral arterial disease in Korean type 2 diabetic patients. *Kidney Blood Press Res*. 2010;33(2):111-118. doi:10.1159/000313594

3. Côté N, Fortier C, Desbiens LC, Nemcsik J, Agharazii M. Individual versus integration of multiple components of central blood pressure and aortic stiffness in predicting cardiovascular mortality in end-stage renal diseases. *J Hum Hypertens*. 2024;38(5):430-436. doi:10.1038/s41371-023-00888-w

4. Guérin AR, Blacher J, Pannier B, et al. Impact of aortic stiffness attenuation on survival of patients in end-stage renal failure. *Circulation*. 2001;103(7):987-992. doi:10.1161/01.CIR.103.7.987

5. Heleniak Z, Illersperger S, Małgorzewicz S, Dębska-Ślizień A, Budde K, Halleck F. Arterial Stiffness as a Cardiovascular Risk Factor After Successful Kidney Transplantation in Diabetic and Nondiabetic Patients. *Transplant Proc*. 2022;54(8):2205-2211. doi:10.1016/j.transproceed.2022.07.007

6. Korogiannou M, Xagas E, Marinaki S, Sarafidis P, Boletis JN. Arterial Stiffness in Patients With Renal Transplantation; Associations With Co-morbid Conditions, Evolution, and Prognostic Importance for Cardiovascular and Renal Outcomes. *Front Cardiovasc Med*. 2019;6:67. doi:10.3389/fcvm.2019.00067

7. Melilli E, Bestard-Matamoros O, Manonelles-Montero A, et al. Arterial stiffness in kidney transplantation: a single center case-control study comparing belatacept versus calcineurin inhibitor immunosuppressive based regimen. *Nefrologia*. 2015;35(1):58-65. doi:10.3265/Nefrologia.pre2014.Sep.12615

8. Moissl-Blanke AP, Delgado GE, Yücel G, et al. Associations between Arterial Stiffness, Electrolytes, and Hormones: Insights from the LURIC Study on Cardiovascular Health. *Cardiorenal Med*. 2025;15(1):659-673. doi:10.1159/000549495

9. Shi J, Ye X, Yu S, et al. Vascular Aging, Cardiovascular-Kidney-Metabolic Syndrome, and Cardiovascular Risk: The Northern Shanghai Study. *JACC Advances*. Published online October 2025:102242. doi:10.1016/j.jacadv.2025.102242

10. Shoji T, Maekawa K, Emoto M, et al. Arterial stiffness predicts cardiovascular death independent of arterial thickness in a cohort of hemodialysis patients. *Atherosclerosis*. 2010;210(1):145-149. doi:10.1016/j.atherosclerosis.2009.11.013

11. Verbeke F, Maréchal C, Van Laecke S, et al. Aortic stiffness and central wave reflections predict outcome in renal transplant recipients. *Hypertension*. 2011;58(5):833-838. doi:10.1161/HYPERTENSIONAHA.111.176594

**Table S2**. Additional baseline characteristics of the included studies and their participants.

| **Reference** | **Tool** | **CKD stages and other characteristics** | **Covariates in adjusted model** | **Length (months)** | **HbA1c (%)** | **Diabetes (%)** | **eGFR (mL/min)** |
| --- | --- | --- | --- | --- | --- | --- | --- |
| Amemiya N et al (2011) | Form/ABI; Omron-Colin Co., Ltd., Komaki, Japan | Stage 5 | Age, sex, presence of diabetes mellitus, time on dialysis, pulse pressure, calcium, phosphorus, intact PTH and haemoglobin levels | 48.0 | NA | 31.7 | NA |
| Avramovski P et al (2014) | Doppler ultrasound (Toshiba SSA-340 A, Toshiba Medical System Co., Tokyo, Japan) | Stage 5 | C-reactive protein and albumin | 36.0 | NA | 20.0 | NA |
| Bao W et al (2019) | Complior (Colson, Gargesles Gonesses, France) | Stage 5 | Age, diastolic pressure, pulse pressure, albumin, diabetes mellitus, eGFR, coronary disease, creatinine | 45.8 | NA | NA | NA |
| Baumann M et al (2014) | Mobil-O-Graph PWA (IEM, Stolberg, Germany) | Stages 2-4 | aPWV, age and mean arterial pressure | 42.0 | NA | 47.0 | 48.4 ± 24.0 |
| Blacher J et al (2003) | Doppler ultrasound (SEGA M842, 10 MHz; Paris, Franceº) | Stage 5 | Age, dialysis length, Haemoglobin | 78.0 | NA | 7.0 | NA |
| Cui X et al (2024) | NA | All: Atherosclerotic heart disease | Sex, body mass index, white blood cell count, serum potassium, serum sodium, blood pH, eGFR, blood urea nitrogen, total bilirubin, triglycerides, LDL, HDL, heart failure, atrial fibrillation, hypertension, diabetes, sepsis, acute kidney injury, use of vasopressin, aspirin, statins, vancomycin, and mechanical ventilation | 12.0 | NA | 53.7 | 55.1 ± 24.6 |
| Eun Yoon H et al (2013) | Colin (VP1000, Colin Co. Ltd, Komaki, Japan) | Stage 2 (60≤eGFR<90 ml/min/1.73 m²): 100  Stage 3a (45≤eGFR<60 ml/min/1.73 m²): 15.  Stage 3b (30≤eGFR<45 ml/min/1.73 m²): 2 | Age, sex, baseline eGFR, change in eGFR, smoking, diabetes, hypertension, statin use, previous cardiovascular disease, systolic and diastolic blood pressure, body mass index, baPWV, haematocrit, fasting glucose, albumin, calcium, phosphorus, total cholesterol, triglycerides, LDL cholesterol, C-reactive protein, and albuminuria | 13.0 | NA | 9.1 | 72.5 ± 11.2 |
| Feng XH et al (2025) | NA | eGFR ≥ 45 mL/min/1,73 m²: 3066  eGFR < 45 mL/min/1,73 m²: 1598 | Sex, age, race/ethnicity, educational level, smoking, hypertension, and the Charlson Comorbidity Index | 75.6 | NA | 28.8 | 47.0 |
| Ferreira JP et al (2017) | Complior (Colson, Les Lilas, France) and Non-directional Doppler (SEGA M842, 10 MHz) | Stage 5 | Left ventricular mass, E/A ratio, age (including the age*PWV interaction term), sex, smoking, albumin, left ventricular ejection fraction, and systolic blood pressure | 74.0 | NA | 9.0 | NA |
| Fortier C et al (2015) | Complior SP (Artech Medical, Pantin, France) and SphygmoCor (AtCor Medical Pty. Ltd, Sydney, Australia | Stage 5 | Age, time on dialysis, sex, previous cardiovascular disease, smoking, diabetes mellitus, and weight | 29.0 | NA | 43.0 | NA |
| Han J et al (2016) | Complior SP (Créatech SAS, Besançon, France) | Stages 2-3 | Age, sex, body mass index, systolic and diastolic blood pressure, fasting glucose, triglycerides, total cholesterol, HDL-C and LDL-C | 57.6 | NA | NA | 84.1 ± 10.5 |
| Kato A et al (2010) | VaSera VS-1000 (Fukuda Denshi, Tokyo, Japan) | Stage 5 | Age, sex, diabetes mellitus | 39.0 | NA | 20.1 | NA |
| Kato A et al (2012) | VeraSera VS-1000 (Fukuda Denshi, Tokyo, Japan) | Stage 5 | Age, sex, diabetes mellitus, time on haemodialysis, current smoking habits, use of ACE inhibitors/ARBs, mean arterial pressure, serum creatinine, calcium, phosphorus, albumin, total cholesterol, haemoglobin, intact PTH, and ferritin | 63.0 | NA | 36.4 | NA |
| Kitahara T et al (2005) | Colin (form PWV/ABI; Colin Medical Technology, Komaki, Japan) | Stage 5 | age, sex, duration of dialysis, diabetes mellitus, smoking history, coronary artery disease, cerebrovascular disease, body mass index (BMI), systolic and diastolic blood pressure, serum albumin levels, calcium-phosphorus product, total cholesterol, Kt/V, haematocrit, ABPI | 33.8 | NA | 33.5 | NA |
| Kuwahara M et al (2013) | Form BP-203RPEIII (Omron-Colin, Tokyo, Japan) | Stage 5 | Age, baseline ABI, rate of ABI decline, baPWV, pro-brain natriuretic peptide, left ventricular ejection fraction, and duration of haemodialysis | 84.0 | 7.0 ± 1.3 | 28.3 | NA |
| Matschkal J et al (2019) | Mobil-O-Graph 24 h-PWA (I.E.M. GmbH, Stolberg, Alemania) | Stage 5 | Age, Charlson Comorbidity Index, calcium-phosphorus product, albumin, dialysis duration | 36.0 | NA | 39.0 | NA |
| Ng NX et al (2023) | SphygmoCor system (AtCor Medical, Australia) | Stage 5 | Age, sex, BMI, diabetes, hypertension, glucose, cholesterol, triglycerides, albumin, BUN, creatinine, and time on dialysis | 36.0 | NA | 40.0 | NA |
| Otsuka K et al (2019) | Colin Waveform Analyzer (form PWV/ABI; Colin Medical Technology, Komai, Japan) | Stage 5 | Age, ABI, left ventricular ejection fraction, pro-brain natriuretic peptide | 43.2 | 5.5 ± 0.9 | 38.0 | NA |
| Shin SJ et al (2009) | PP-1000 Applanation Tonometer (Hanbyul Meditech Co., based in Seoul, South Korea) | Stage 5 | Age, gender, time on dialysis, diabetes, and mean systolic blood pressure | 46.0 | NA | 45.8 | NA |
| Tanaka M et al (2011) | Colin waveform analyser (form PWV/ABI; Colin Medical Technology, Komaki, Japan) | Stage 5 | Age, the presence of diabetes, and a history of previous coronary artery disease | 43.0 | NA | 47.5 | NA |
| Tripepi G et al (2018) | Transcutaneous Doppler (SEGA M842 Doppler unit, 10 MHz) and Gould 8188 recorder, and Complior SP (Artech Medical, Pantin, France) | Stage 5 | Age, smoking, BMI, history of CV disease, history of cancer, aetiology of CKD, type of vascular access, blood flow, haemoglobin, ferritin, C-reactive protein, serum albumin, and creatinine | 24 | NA | 27.0 | NA |
| Townsend RR et al (2018) | Sphygmocor PVx System (AtCor Medical, Sydney, Australia) | Stages 3-4 | Mean arterial pressure, age, sex, race/ethnicity, proteinuria, baseline eGFR, and clinical study center | 64.8 | 6.9 ± 1.5 | 47.3 | 44.4 ± 18.2 |
| Verbeke F et al (2011) | SphygmoCor v7 (AtCor Medical, Sydney, Australia | Stage 5 | Age, diabetes mellitus, and serum albumin levels | 24 | NA | NA | NA |
| Wang LL et al (2018) | Vascular profiler Bp-203RPEIII (Omron Co. Ltd, China) | Stage 5 | sex, age, body mass index, blood pressure, haemoglobin, serum albumin, calcium, phosphorus, intact parathyroid hormone, uric acid and medication use (ACE inhibitors, ARBs) | 31.5 | NA | 22.4 | NA |
| Wang Y et al (2026) | Complior SP (Artech-Medical, France) | Stage 3a:30  Stage 3b: 24  Stage 4: 38  Stage 5: 141 | Age, sex, history of cardiovascular disease, diabetes mellitus, systolic and diastolic blood pressure, type of antihypertensive medication, dialysis status, proteinuria, haemoglobin, eGFR, and albumin | 108 | NA | 40.8 | 18.8 ± 16.2 |
| Xu T et al (2015) | SphygmoCor pulse wave analysis (AtCor Medical, West Ryde, Australia) | Stage 5 | Age, gender, dialysis duration, BMI, hs-CRP, iPTH, haemoglobin (Hb), blood pressure (BP), left atrial diameter (LAD), ejection fraction (EF), left ventricular mass index (LVMI), dialysis adequacy, and nPCR | 21.2 | NA | NA | 4.0 ± 2.3 |
| Zang H et al (2026) | NA | NA | Sex, age, race, body mass index, smoking, alcohol consumption, history of cardiovascular events, hypertension, anaemia, and type 2 diabetes | 120 | NA | 35.0 | 72.6 ± 28.8 |
| Zoungas S et al (2007) | Tonometer Millar Mikro-tip (SPT-301; Millar Instruments, Houston, Texas, US) | Stages 4-5 | Age, sex, systolic blood pressure, diastolic blood pressure, diabetes, history of previous cardiovascular disease, total cholesterol levels, and smoking | 43.2 | NA | 25.1 | NA |

Abbreviations: ABPI – ankle–brachial pressure index; ABI – ankle–brachial index; ACE inhibitors/ARBs – angiotensin-converting enzyme inhibitors/angiotensin receptor blockers; aPWV – aortic Pulse Wave Velocity; baPWV – brachial-ankle Pulse Wave Velocity; BMI – body mass index; BUN – blood urea nitrogen; CKD – chronic kidney disease; CV disease – cardiovascular disease; e/a ratio – early to late diastolic mitral inflow velocity ratio; eGFR – estimated glomerular filtration rate; HbA1c – glycated hemoglobin; HDL – high-density lipoprotein; HDL-C – high-density lipoprotein cholesterol; HR: hazard ratio; hs-CRP – high-sensitivity C-reactive protein; Kt/V – dialysis adequacy index; LDL – low-density lipoprotein; LDL-C – low-density lipoprotein cholesterol; NA: not available; nPCR – normalized protein catabolic rate; PTH – parathyroid hormone; NA: not available.

**Table S3.** Quality assessment of cohorts and cross-sectional studies.

| **Reference** | **1** | **2** | **3** | **4** | **5** | **6** | **7** | **8** | **9** | **10** | **11** | **12** | **13** | **14** | **Overall** |
| --- | --- | --- | --- | --- | --- | --- | --- | --- | --- | --- | --- | --- | --- | --- | --- |
| Amemiya N et al (2011) | Yes | Yes | Yes | Yes | No | Yes | Yes | Yes | Yes | SC | Yes | SC | Yes | Yes | Good |
| Avramovski P et al (2014) | Yes | Yes | SC | Yes | No | Yes | Yes | Yes | Yes | SC | Yes | SC | Yes | Yes | Good |
| Bao W et al (2019) | Yes | Yes | SC | Yes | No | Yes | Yes | Yes | Yes | SC | Yes | SC | Yes | Yes | Good |
| Baumann M et al (2014) | Yes | Yes | SC | Yes | No | Yes | Yes | Yes | Yes | SC | Yes | SC | Yes | Yes | Good |
| Blacher J et al (2003) | Yes | Yes | SC | Yes | No | Yes | Yes | Yes | Yes | SC | Yes | SC | Yes | Yes | Good |
| Cui X et al (2024) | Yes | Yes | No | Yes | No | Yes | Yes | Yes | Yes | SC | Yes | SC | Yes | Yes | Fair |
| Eun Yoon H et al (2013) | Yes | Yes | No | Yes | No | Yes | Yes | Yes | Yes | SC | Yes | SC | No | Yes | Poor |
| Feng XH et al (2025) | Yes | Yes | SC | Yes | SC | Yes | Yes | Yes | Yes | SC | Yes | Yes | Yes | Yes | Good |
| Ferreira JP et al (2017) | Yes | Yes | SC | Yes | SC | Yes | Yes | Yes | Yes | SC | Yes | SC | Yes | Yes | Good |
| Fortier C et al (2015) | Yes | Yes | Yes | Yes | No | Yes | Yes | Yes | Yes | SC | Yes | SC | Yes | Yes | Good |
| Han J et al (2016) | Yes | Yes | Yes | Yes | No | Yes | Yes | Yes | Yes | SC | Yes | SC | Yes | Yes | Good |
| Kato A et al (2010) | Yes | Yes | No | Yes | No | Yes | Yes | Yes | Yes | SC | Yes | SC | Yes | Yes | Fair |
| Kato A et al (2012) | Yes | Yes | Yes | Yes | No | Yes | Yes | Yes | Yes | SC | Yes | SC | Yes | Yes | Good |
| Kitahara T et al (2005) | Yes | Yes | Yes | Yes | No | Yes | Yes | Yes | Yes | SC | Yes | SC | Yes | Yes | Good |
| Kuwahara M et al (2013) | Yes | Yes | Yes | Yes | No | Yes | Yes | Yes | Yes | Yes | Yes | SC | Yes | Yes | Good |
| Matschkal J et al (2019) | Yes | Yes | Yes | Yes | No | Yes | Yes | Yes | Yes | SC | Yes | SC | Yes | Yes | Good |
| Ng NX et al (2023) | Yes | Yes | Yes | Yes | No | Yes | Yes | Yes | Yes | SC | Yes | SC | Yes | Yes | Good |
| Otsuka K et al (2019) | Yes | Yes | SC | Yes | No | Yes | Yes | Yes | Yes | SC | Yes | Yes | Yes | Yes | Good |
| Shin SJ et al (2009) | Yes | Yes | Yes | Yes | No | Yes | Yes | Yes | Yes | SC | Yes | Yes | Yes | Yes | Good |
| Tanaka M et al (2011) | Yes | Yes | Yes | Yes | No | Yes | Yes | Yes | Yes | SC | Yes | SC | Yes | Yes | Good |
| Tripepi G et al (2018) | Yes | Yes | Yes | Yes | No | Yes | Yes | Yes | Yes | SC | Yes | SC | Yes | Yes | Good |
| Townsend RR et al (2018) | Yes | Yes | Yes | Yes | SC | Yes | Yes | Yes | Yes | SC | Yes | SC | Yes | Yes | Good |
| Verbeke F et al (2011) | Yes | Yes | SC | Yes | No | Yes | Yes | Yes | Yes | SC | Yes | SC | Yes | Yes | Good |
| Wang LL et al (2018) | Yes | Yes | SC | Yes | No | Yes | Yes | Yes | Yes | SC | Yes | SC | Yes | Yes | Good |
| Wang Y et al (2026) | Yes | Yes | Yes | Yes | No | Yes | Yes | Yes | Yes | SC | Yes | SC | Yes | Yes | Good |
| Xu T et al (2015) | Yes | Yes | Yes | Yes | No | Yes | Yes | Yes | Yes | SC | Yes | SC | Yes | Yes | Good |
| Zang H et al (2026) | Yes | Yes | SC | Yes | SC | Yes | Yes | Yes | Yes | SC | Yes | SC | SC | Yes | Good |
| Zoungas S et al (2007) | Yes | Yes | SC | Yes | SC | Yes | Yes | Yes | Yes | Yes | Yes | Yes | Yes | Yes | Good |

Assessment of risk of bias for observational studies with 'Study Quality Assessment Tools'. Green: yes (Y); Red: no (N); Yellow: Some concerns, including not determine, not reported and not applicable.

**Items for Study Quality Assessment Tools:**

1. Was the research question or objective in this paper clearly stated?
2. Was the study population clearly specified and defined?
3. Was the participation rate of eligible persons at least 50%?
4. Were all the subjects selected or recruited from the same or similar populations (including the same time period)? Were inclusion and exclusion criteria for being in the study prespecified and applied uniformly to all participants?
5. Was a sample size justification, power description, or variance and effect estimates provided?
6. For the analyses in this paper, were the exposure(s) of interest measured prior to the outcome(s) being measured?
7. Was the timeframe sufficient so that one could reasonably expect to see an association between exposure and outcome if it existed?
8. For exposures that can vary in amount or level, did the study examine different levels of the exposure as related to the outcome (e.g., categories of exposure, or exposure measured as continuous variable)?
9. Were the exposure measures (independent variables) clearly defined, valid, reliable, and implemented consistently across all study participants?
10. Was the exposure(s) assessed more than once over time?
11. Were the outcome measures (dependent variables) clearly defined, valid, reliable, and implemented consistently across all study participants?
12. Were the outcome assessors blinded to the exposure status of participants?
13. Was loss to follow-up after baseline 20% or less?
14. Were key potential confounding variables measured and adjusted statistically for their impact on the relationship between exposure(s) and outcome(s)?

**Table S4.** Quality of evidence assessment.

| Certainty assessment | | | | | | | Effect Size | Certainty |
| --- | --- | --- | --- | --- | --- | --- | --- | --- |
| № of studies | **Study design** | **Risk of bias** | **Inconsistency** | **Indirectness** | **Imprecision** | **Other considerations** |  |  |
| 1. All-cause mortality – aPWV Categorical | | | | | | | | |
| 5 | Observational | Not serious | Very serious | Not serious | Serious | Publication bias | HR = 3.83 (1.19, 12.37) | Very low |
| 1. All-cause mortality – aPWV Linear | | | | | | | | |
| 10 | Observational | Not serious | Very serious | Not serious | Not serious | Publication bias | HR = 1.16 (1.06, 1.28) | Very low |
| 1. All-cause mortality – baPWV Categorical | | | | | | | | |
| 6 | Observational | Not serious | Not serious | Not serious | Not serious | None | HR = 1.99 (1.35, 2.95) | Low |
| 1. All-cause mortality – ePWV Categorical | | | | | | | | |
| 4 | Observational | Not serious | Very serious | Not serious | Not serious | None | HR = 2.22 (1.34, 3.68) | Very low |
| 1. Cardiovascular mortality – aPWV Categorical | | | | | | | | |
| 2 | Observational | Not serious | Serious | Not serious | Serious | None | HR = 23.63 (1.18, 474.79) | Very low |
| 1. Cardiovascular mortality – aPWV Linear | | | | | | | | |
| 5 | Observational | Not serious | Very serious | Not serious | Not serious | Publication bias | HR = 1.36 (1.15, 1.61) | Very low |
| 1. Cardiovascular mortality – baPWV Categorical | | | | | | | | |
| 3 | Observational | Not serious | Not serious | Not serious | Serious | None | HR = 4.28 (1.45, 12.63) | Very low |
| 1. Cardiovascular mortality – ePWV Categorical | | | | | | | | |
| 2 | Observational | Not serious | Not serious | Not serious | Not serious | None | HR = 2.03 (1.06, 3.87) | Low |
| 1. Cardiovascular disease – aPWV Linear | | | | | | | | |
| 4 | Observational | Not serious | Very serious | Not serious | Not serious | Publication bias | HR = 1.20 (1.02, 1.41) | Very low |
| 1. Cardiovascular disease – baPWV Linear | | | | | | | | |
| 4 | Observational | Not serious | Not serious | Not serious | Not serious | None | HR = 1.37 (1.09, 1.72) | Low |

Abbreviations: aPWV – aortic Pulse Wave Velocity, baPWV – brachial-ankle Pulse Wave Velocity; ePWV – estimated Pulse Wave Velocity; HR: hazard ratio; nº: number.[

**Table S5.** Publication bias and Trim-and-Fill test.

| **Outcome** | **Egger test** | **Trim-and-fill test** | |
| --- | --- | --- | --- |
|  |  | **Before imputing** | **After imputing** |
| **All-cause mortality** | | | |
| aPWV – Categorical | p = 0.003 | 3.83 (1.19, 12.37), n = 5 | 3.83 (1.19, 12.37), n = 5 |
| aPWV – Linear | p = 0.007 | 1.16 (1.06, 1.28), n = 10 | 1.13 (0.99, 1.28), n = 12 |
| baPWV – Categorical | p = 0.663 | 1.99 (1.35, 2.95), n = 6 | 1.71 (1.18, 2.49), n = 8 |
| ePWV - Categorical | p = 0.593 | 2.22 (1.34, 3.68), n = 4 | 2.22 (1.34, 3.68), n = 4 |
| **Cardiovascular mortality** | | | |
| aPWV – Linear | p = 0.001 | 1.36 (1.15, 1.61), n = 5 | 1.36 (1.15, 1.61), n = 5 |
| baPWV – Categorical | p = 0.277 | 4.28 (1.45, 12.63), n = 3 | 4.28 (1.45, 12.63), n = 3 |
| **Cardiovascular disease** | | | |
| aPWV – Linear | p = 0.025 | 1.20 (1.02, 1.41), n = 4 | 1.11 (0.92, 1.35), n = 6 |
| baPWV – Categorical | p = 0.771 | 1.37 (1.09, 1.72), n = 4 | 1.46 (1.17, 1.81), n = 5 |

Abbreviations: aPWV – aortic Pulse Wave Velocity, baPWV – brachial-ankle Pulse Wave Velocity; ePWV – estimated Pulse Wave Velocity.

**Table S6.** Metaregressions analyses.

| **Covariate** | **Age** | **Females** | **Diabetes** | **Length** |
| --- | --- | --- | --- | --- |
| **All-cause mortality** | | | | |
| aPWV – Categorical | -0.5233 (p = 0.003) | -3.7927 (p = 0.710) | -5.9381 (p = 0.050) | 0.0104 (p = 0.826) |
| aPWV – Linear | -0.0060 (p = 0.540) | -1.0246 (p = 0.332) | -0.3969 (p = 0.233) | 0.0003 (p = 0.870) |
| baPWV – Categorical | -0.0521 (p = 0.707) | 9.0821 (p = 0.287) | 0.6586 (p = 0.731) | -0.0077 (p = 0.843) |
| ePWV - Categorical | NA | 2.9679 (p = 0.083) | -3.7489 (p = 0.083) | 0.0066 (p = 0.083) |
| **Cardiovascular mortality** | | | | |
| aPWV – Linear | -0.0007 (p = 0.960) | -0.2925 (p = 0.859) | -0.0747 (p = 0.911) | -0.0002 (p = 0.962) |
| baPWV – Categorical | -0.5331 (p = 0.274) | -19.9903 (p = 0.274) | -13.3269 (p = 0.274) | 0.0799 (p = 0.274) |
| **Cardiovascular disease** | | | | |
| aPWV – Linear | -0.0135 (p = 0.328) | 0.6591 (p = 0.585) | 0.1666 (0.845) | 0.0065 (p = 0.222) |
| baPWV – Categorical | -0.0187 (p = 0.672) | 0.0273 (p = 0.996) | -0.2498 (p = 0.870) | -0.0447 (p = 0.687) |

Abbreviations: aPWV – aortic Pulse Wave Velocity, baPWV – brachial-ankle Pulse Wave Velocity; ePWV – estimated Pulse Wave Velocity.

**Figure S1.** Publication bias assessment for the risk of all-cause mortality.

1. aPWV – Categorical.


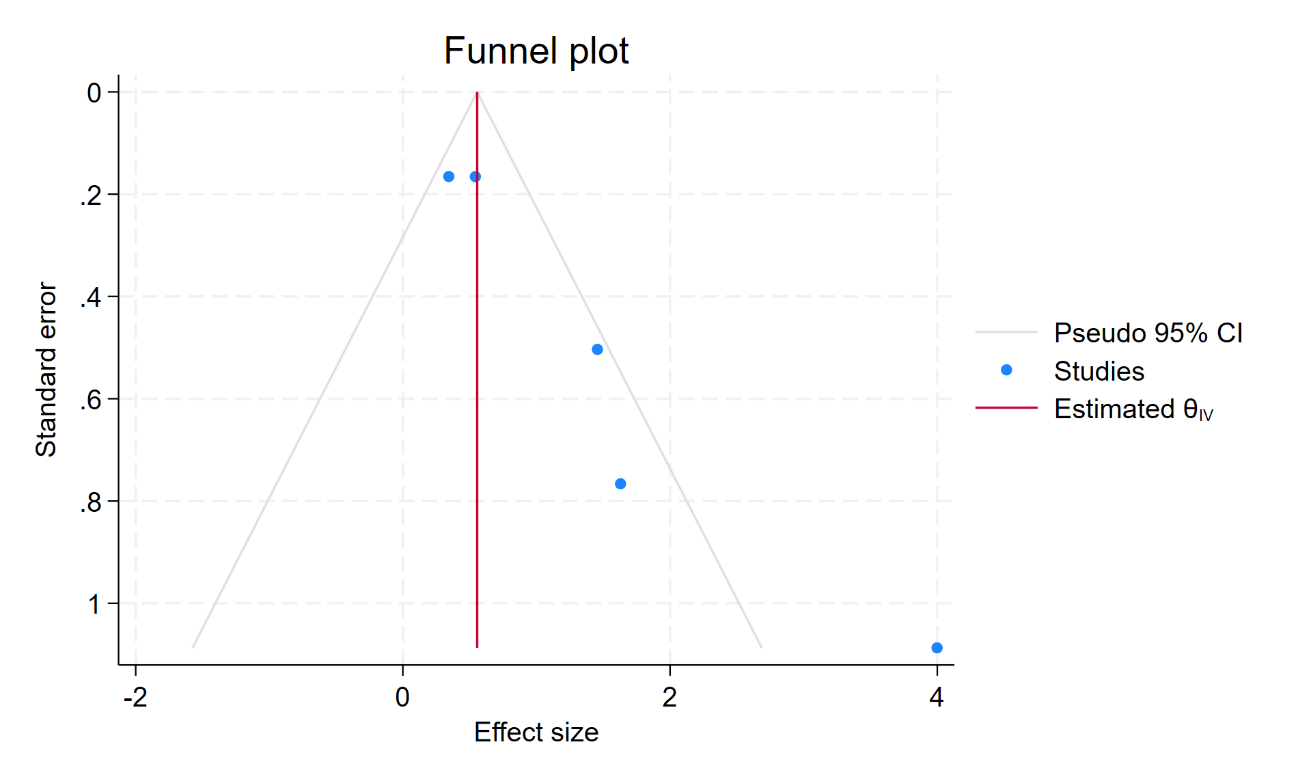


1. aPWV – Linear.


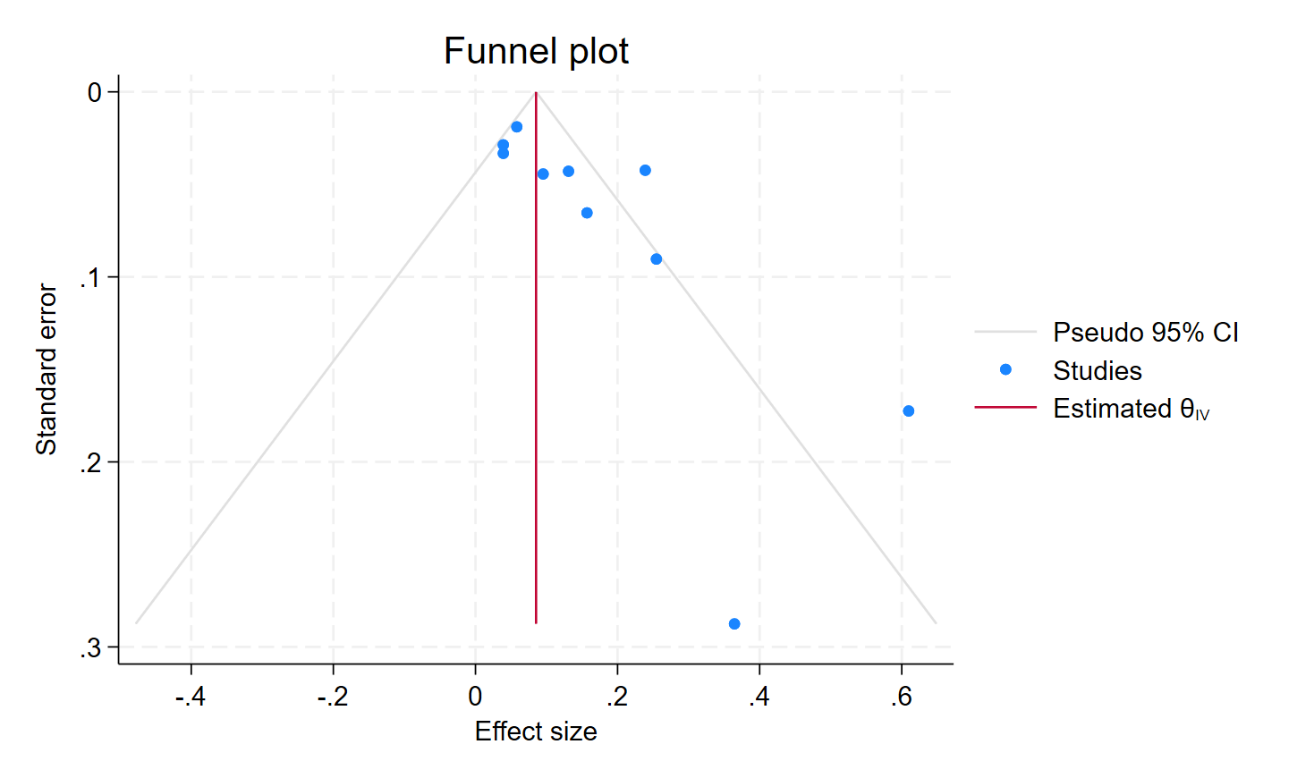


1. baPWV – Categorical.


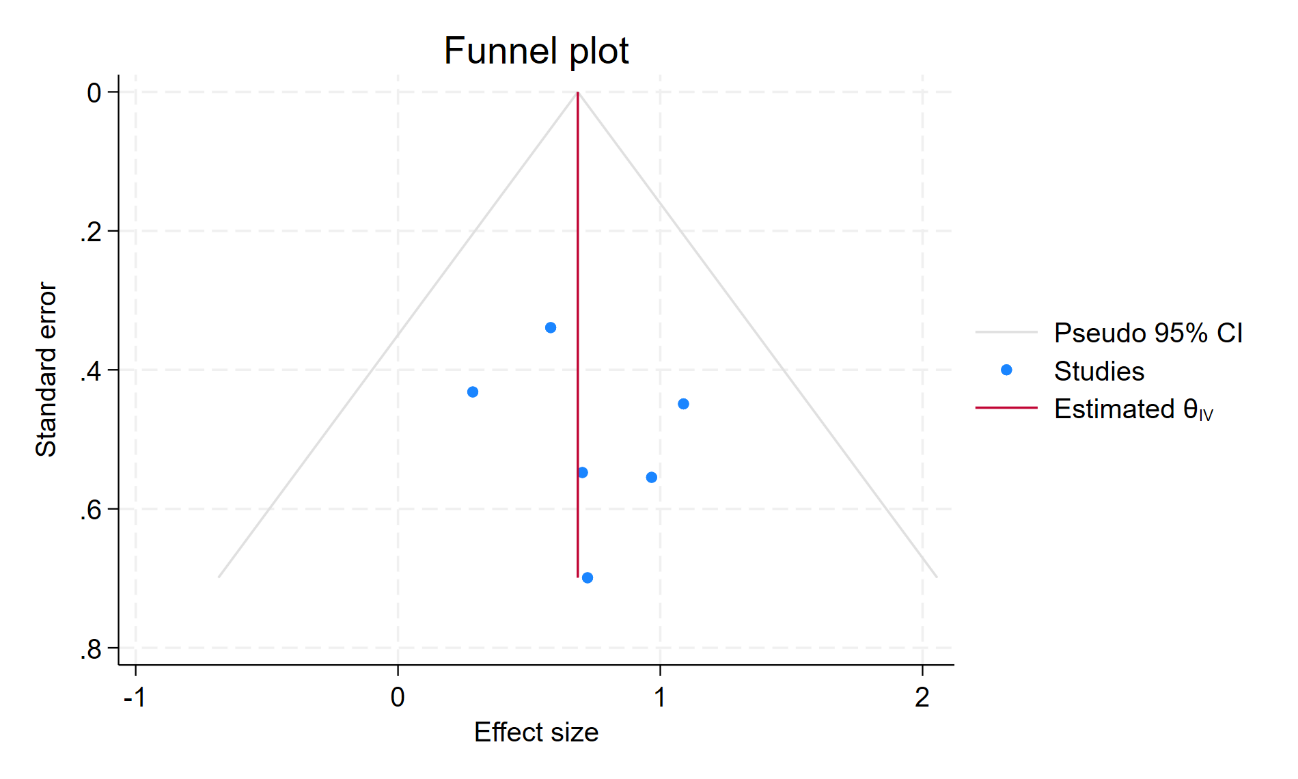


1. ePWV – Categorical.


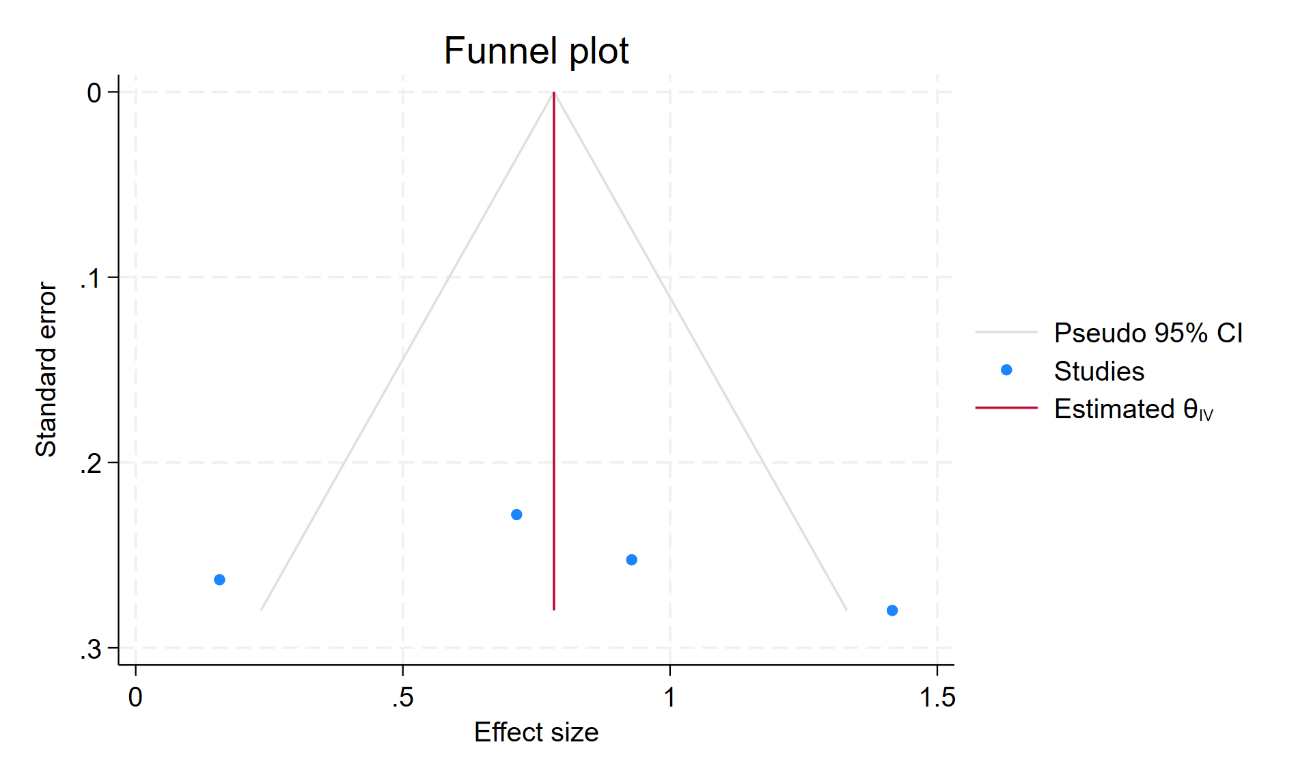


**Figure S2.** Publication bias assessment for the risk of cardiovascular mortality.

1. aPWV – Linear.


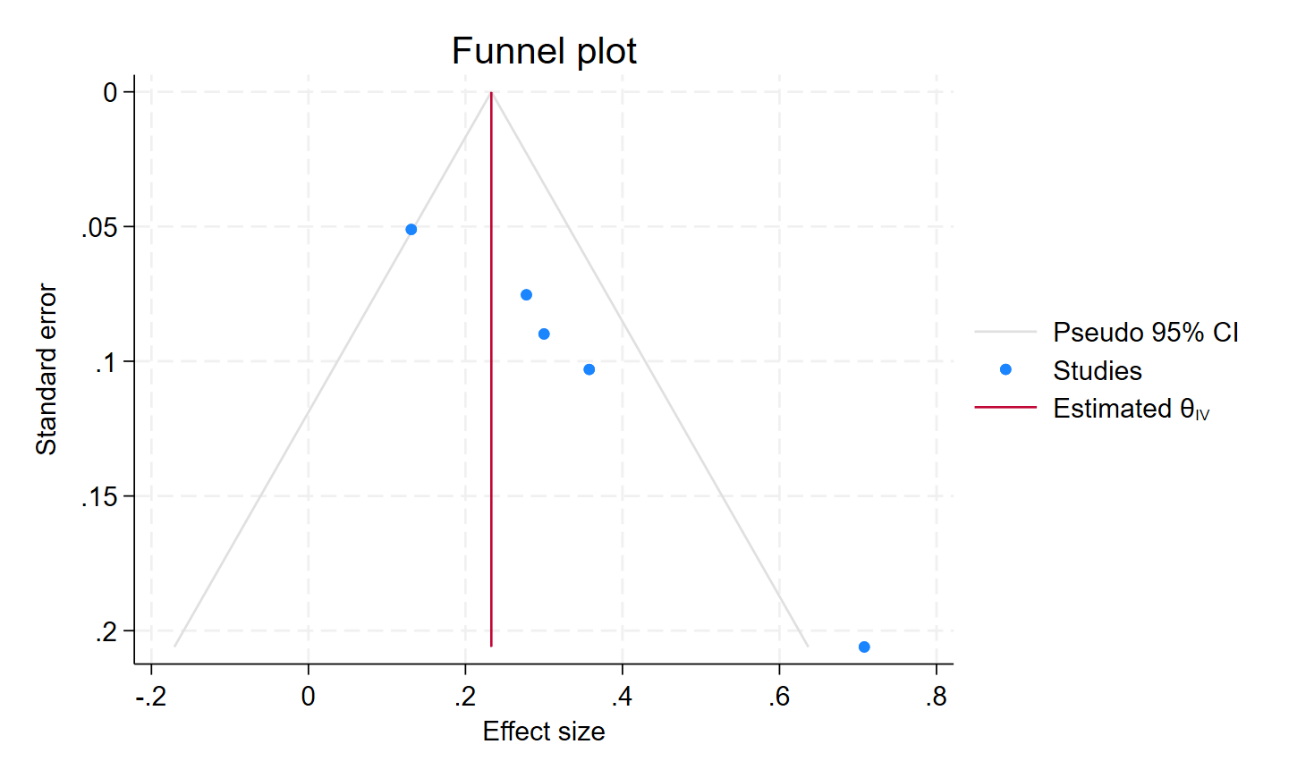


1. baPWV – Categorical.


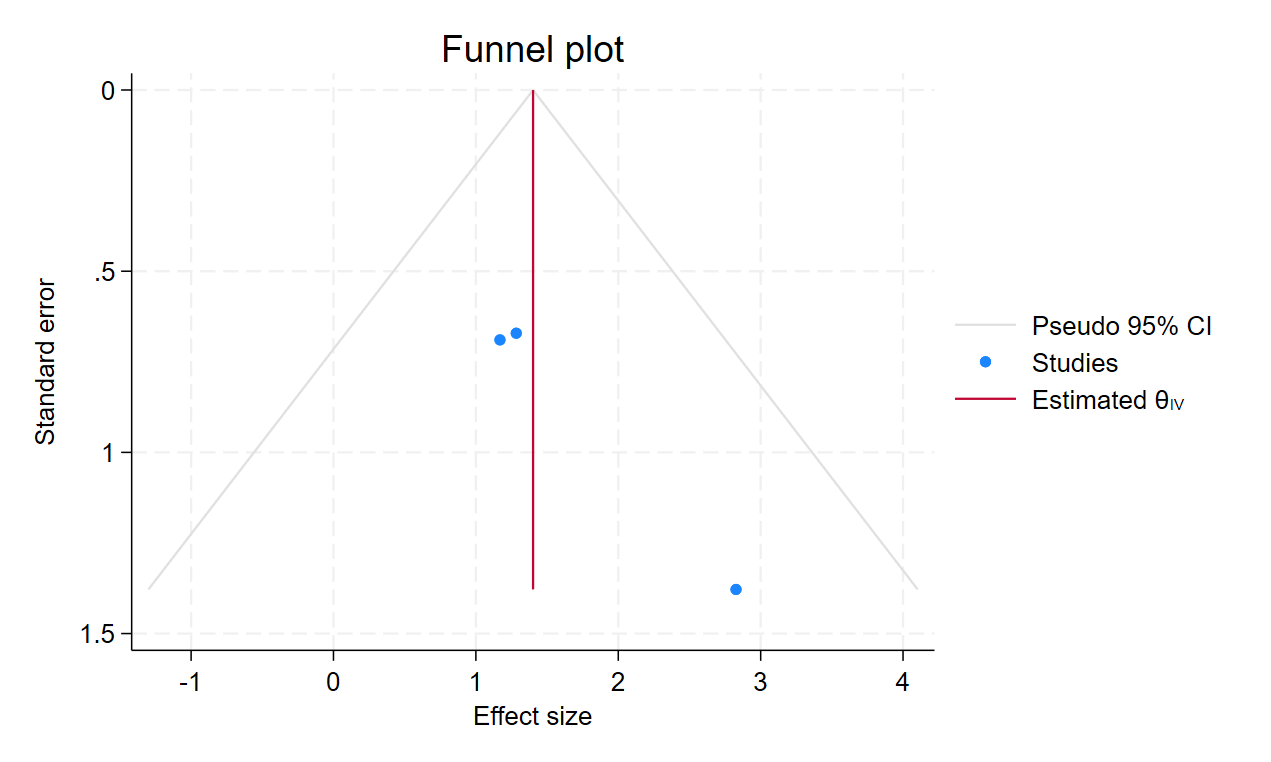


**Figure S3.** Publication bias assessment for the risk of cardiovascular disease.

1. aPWV – Linear.


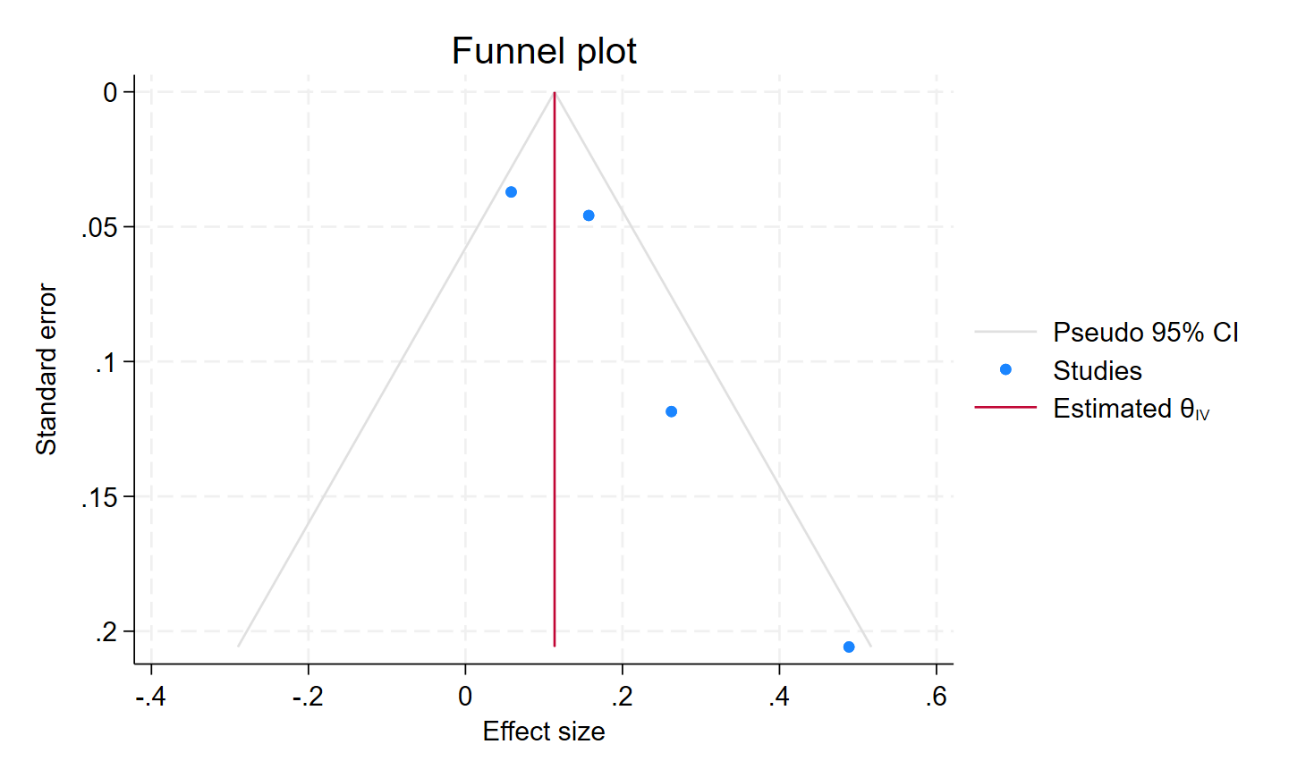


1. baPWV – Categorical.


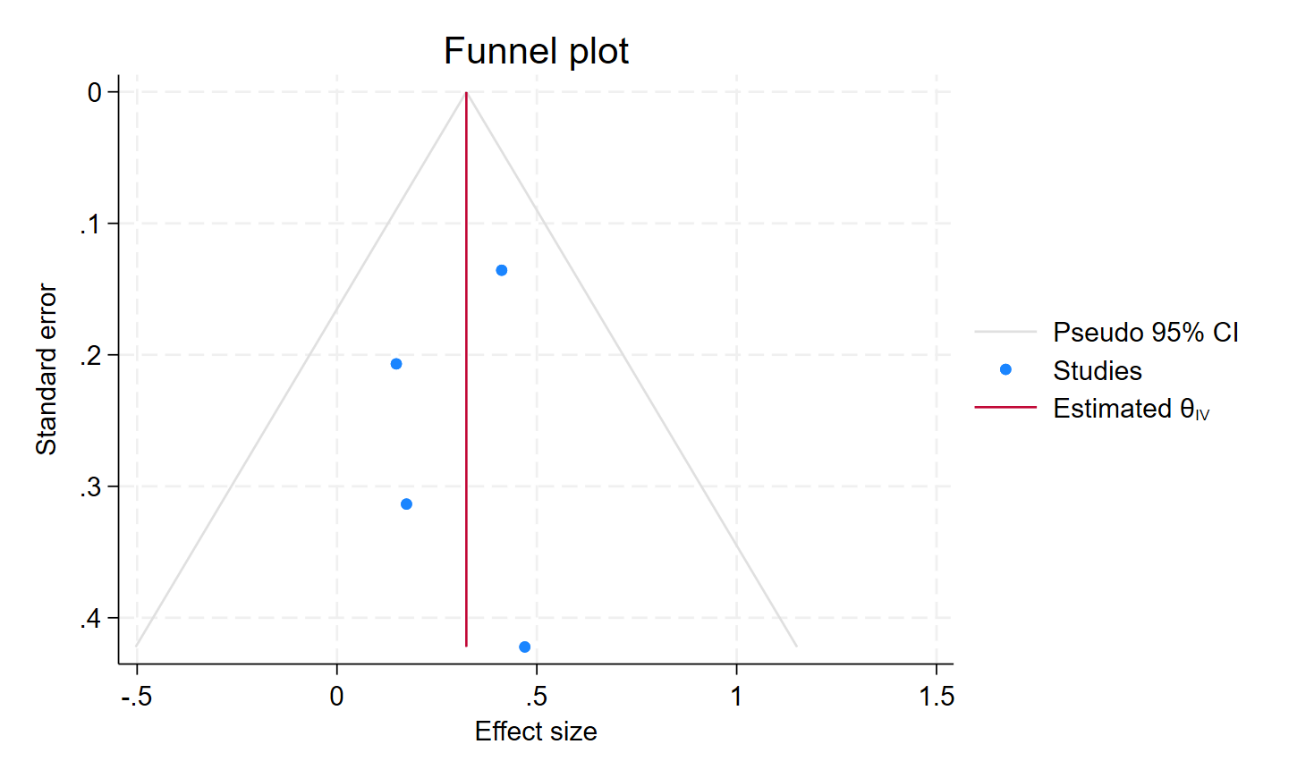


**Figure S4.** Sensitivity analyses for the risk of all-cause mortality.

1. aPWV – Categorical.


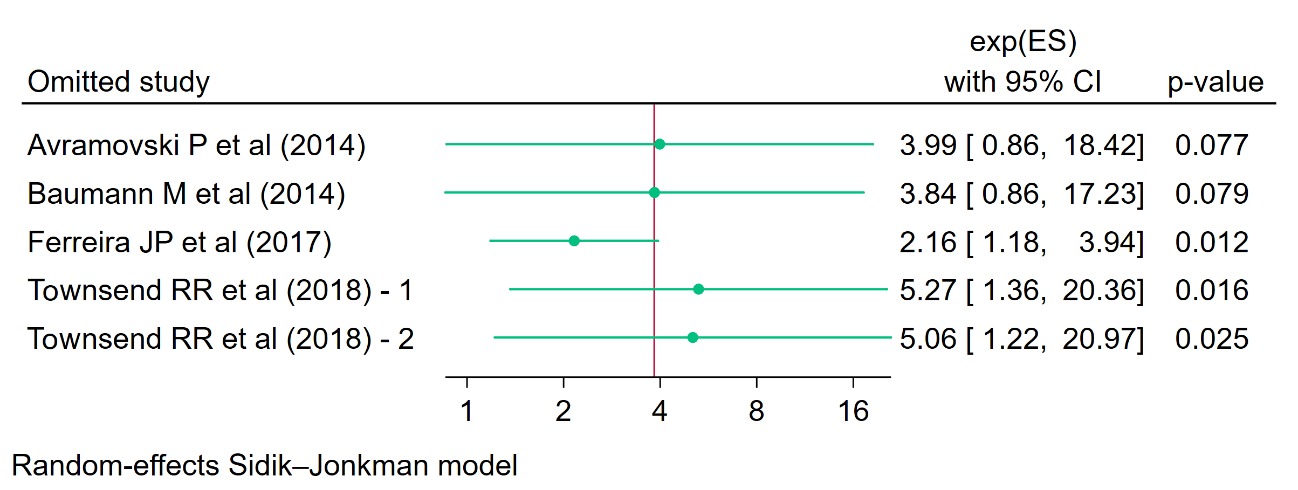


1. aPWV – Linear.


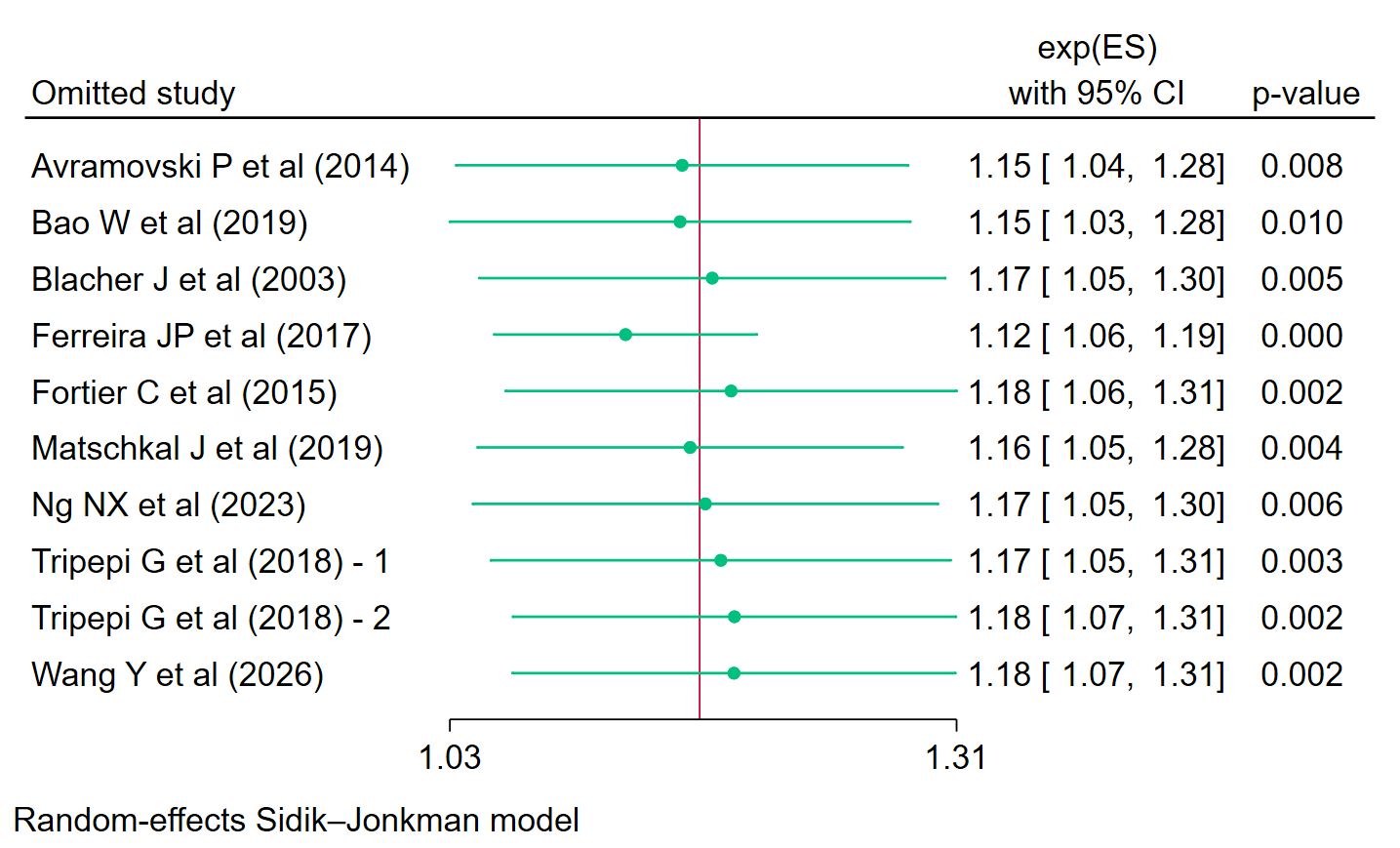


1. baPWV – Categorical.


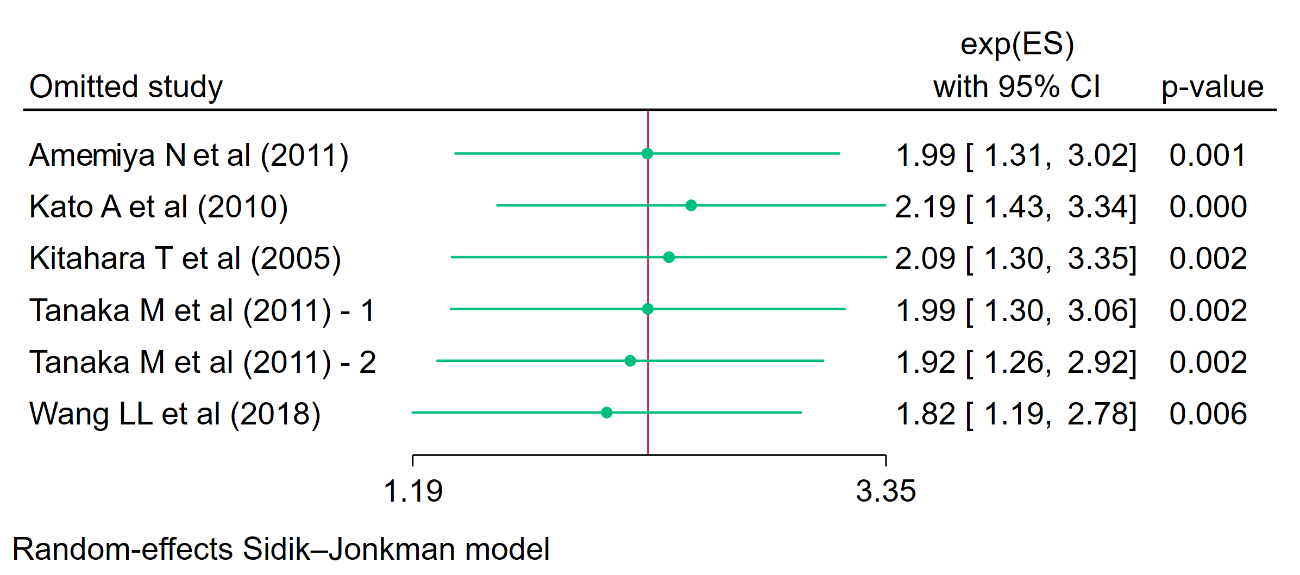


1. ePWV – Categorical.


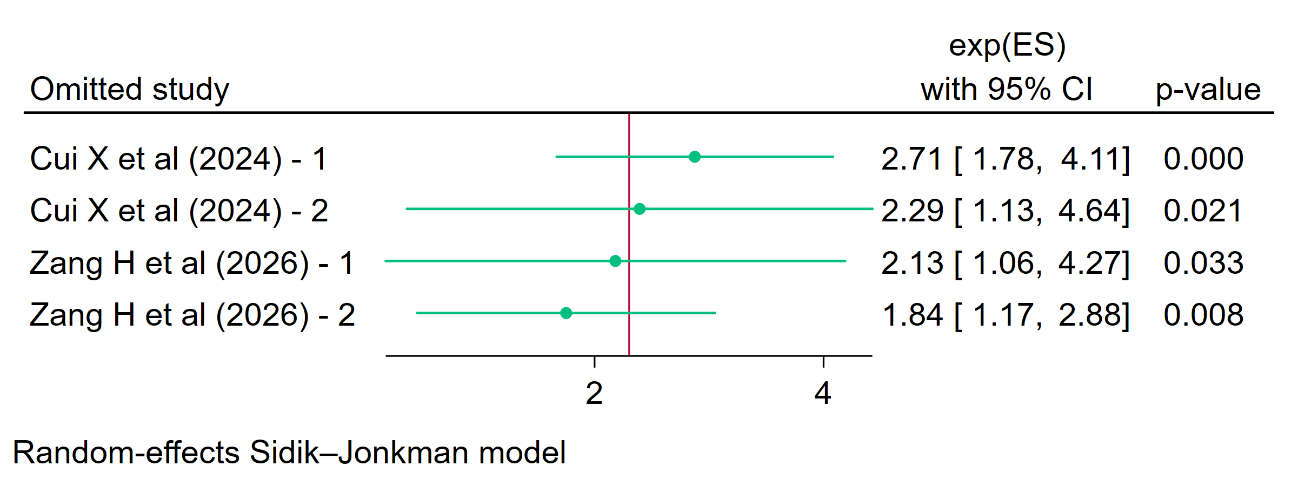


**Figure S5.** Sensitivity analyses for the risk of cardiovascular mortality.

1. aPWV – Linear.


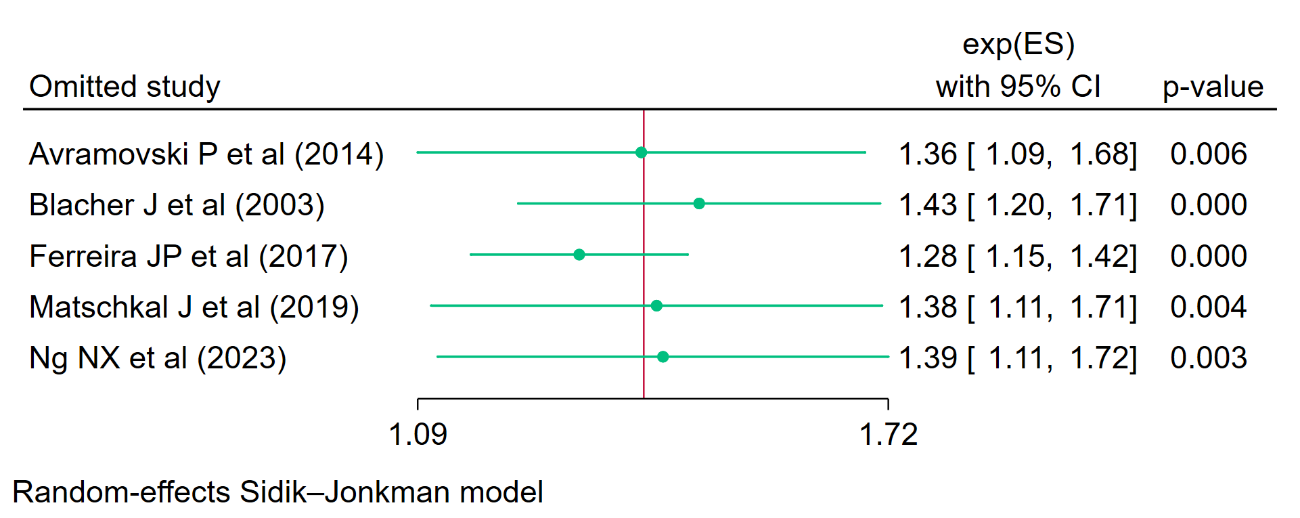


1. baPWV – Categorical.


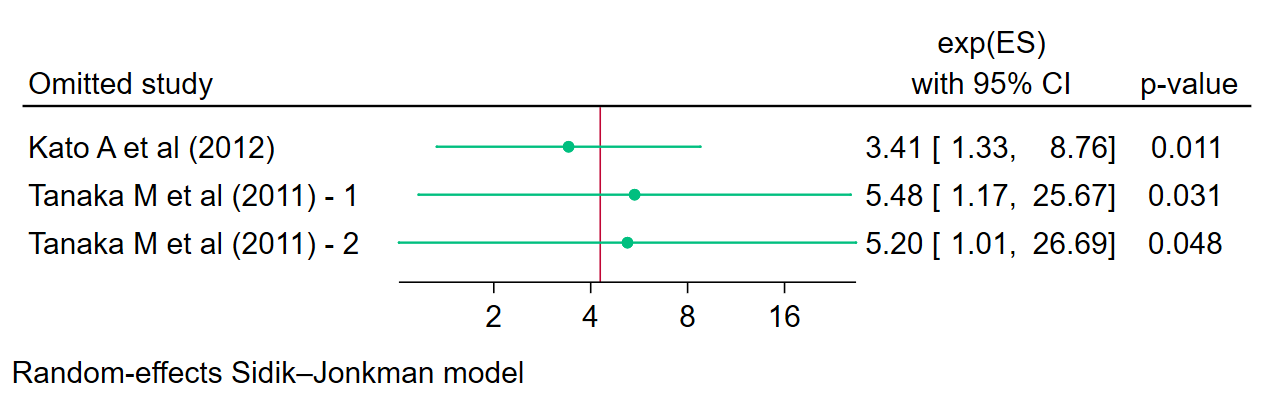


**Figure S6.** Sensitivity analyses for the risk of cardiovascular disease.

1. aPWV – Linear.


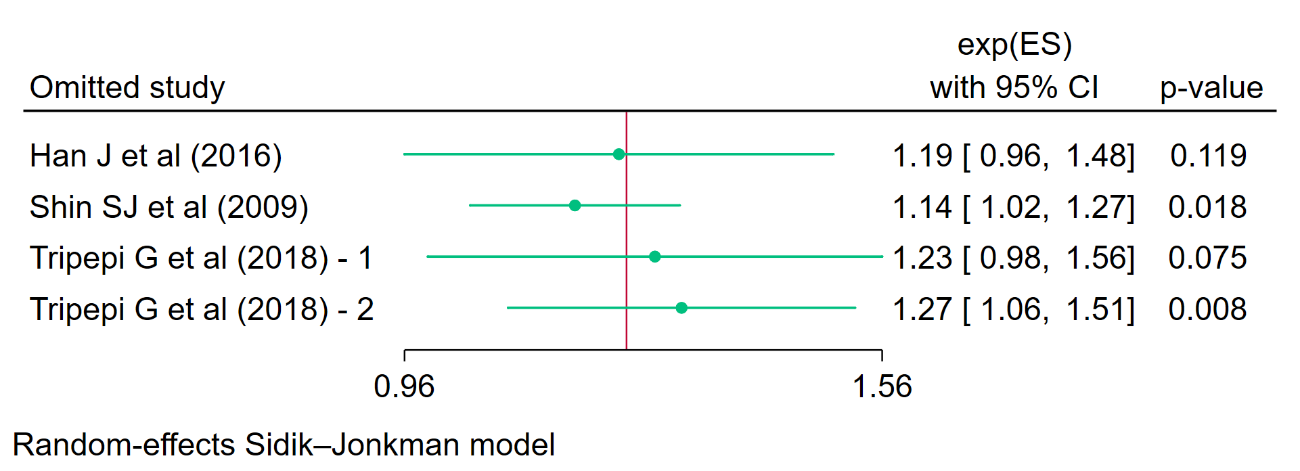


1. baPWV – Categorical.


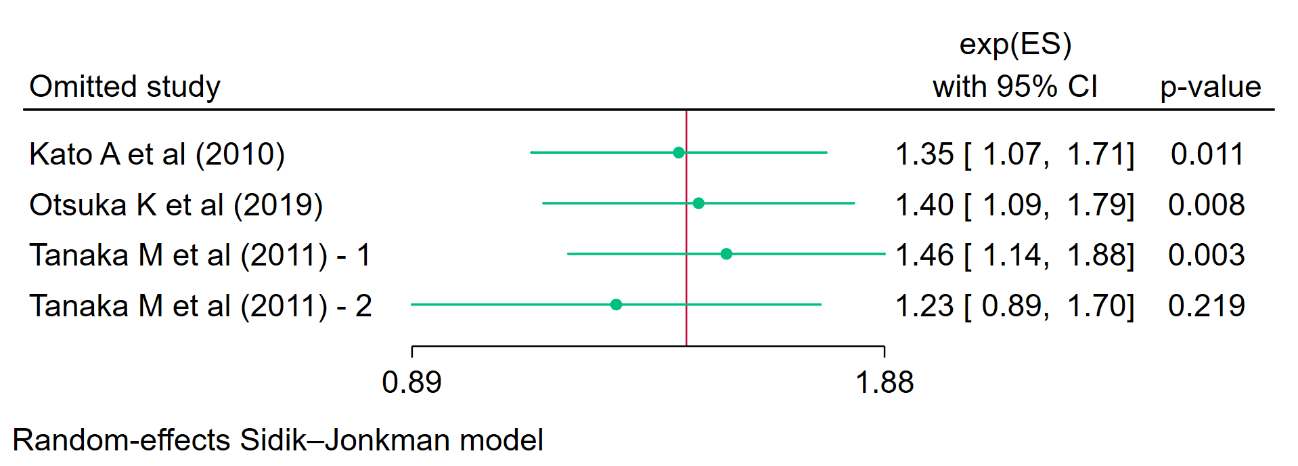


**Figure S7.** Galbraith plots for the risk of all-cause mortality.

1. aPWV – Categorical.


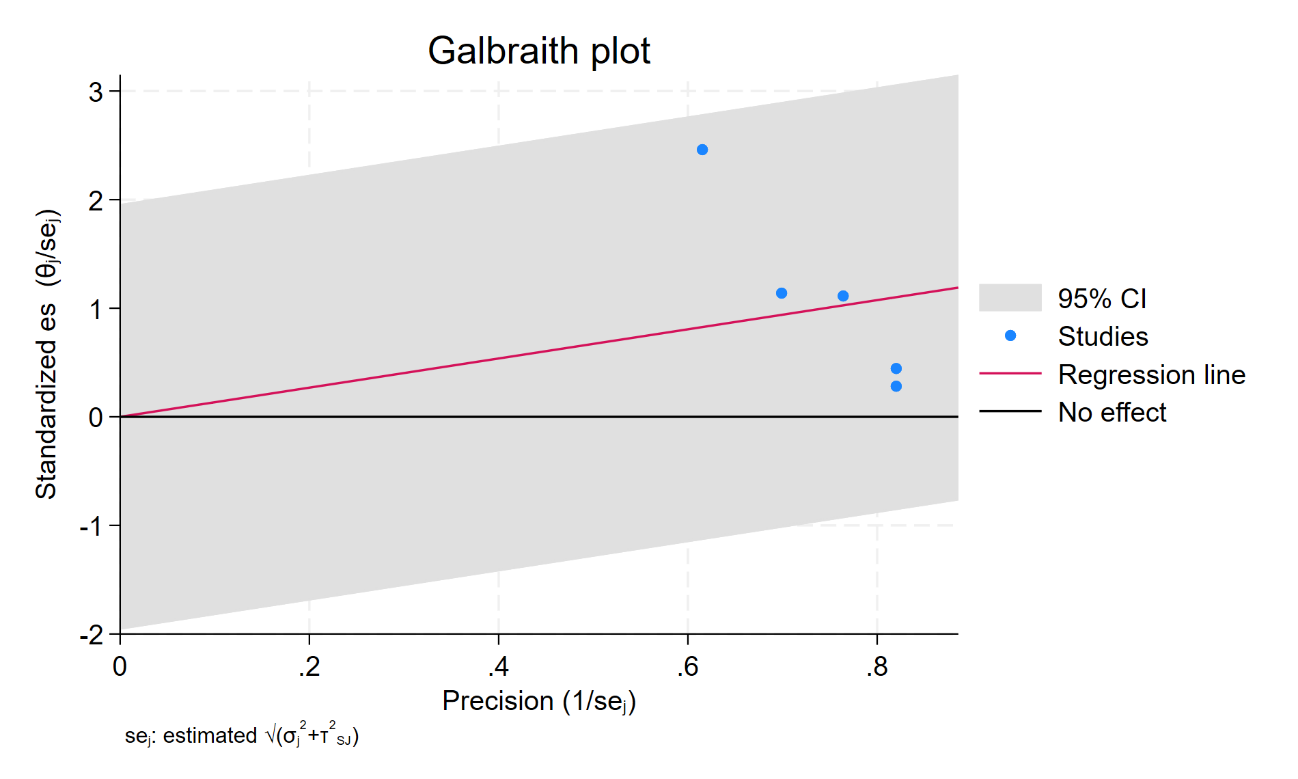


1. aPWV – Linear.


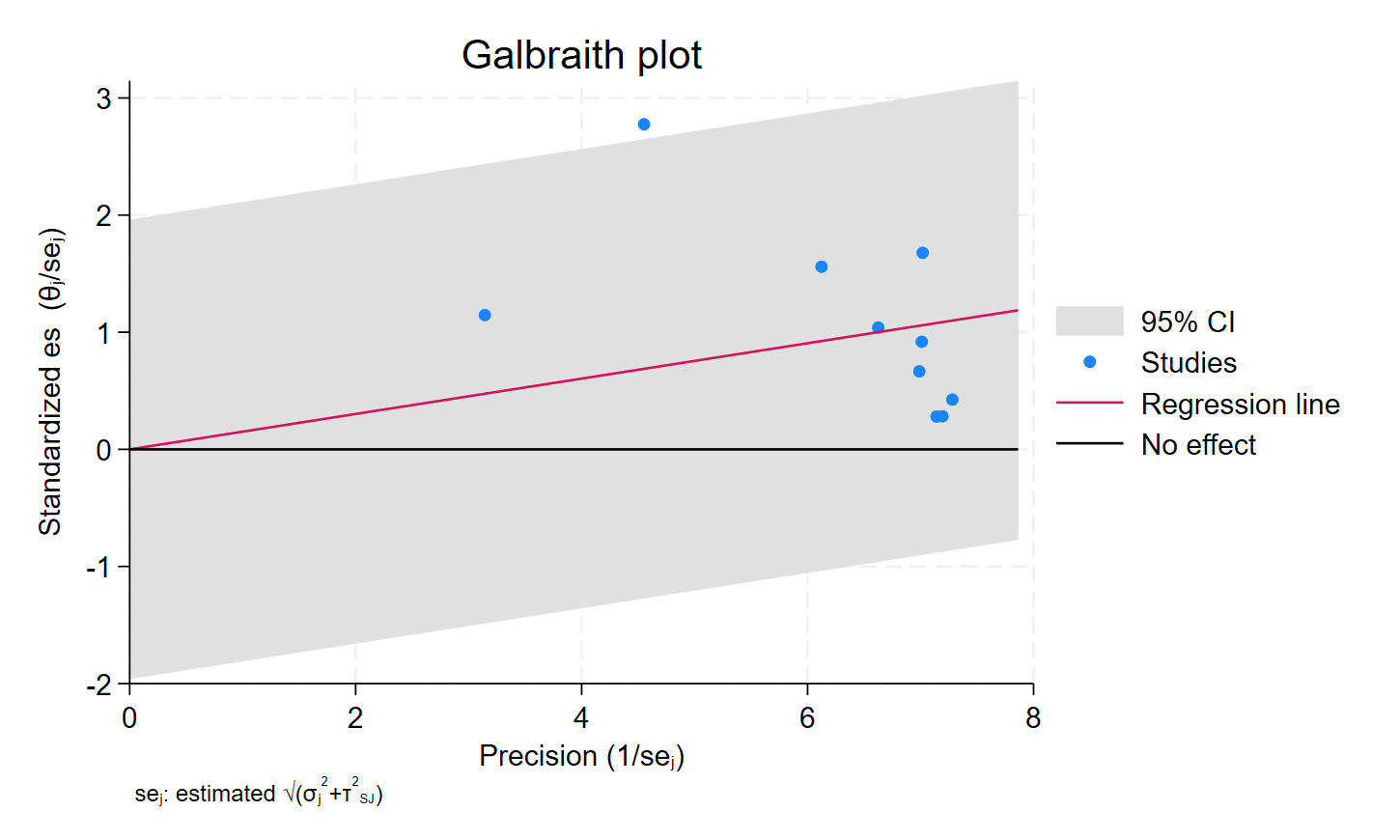


1. baPWV – Categorical.


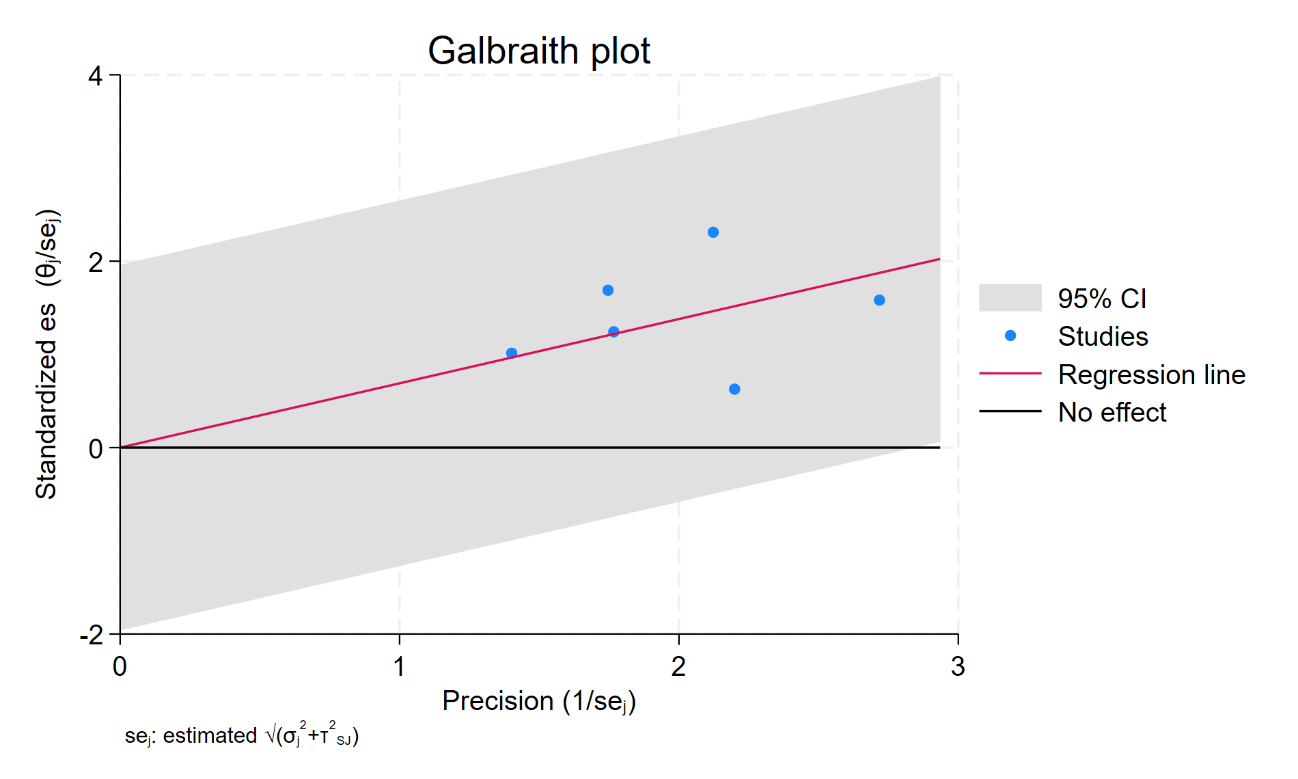


1. ePWV – Categorical.


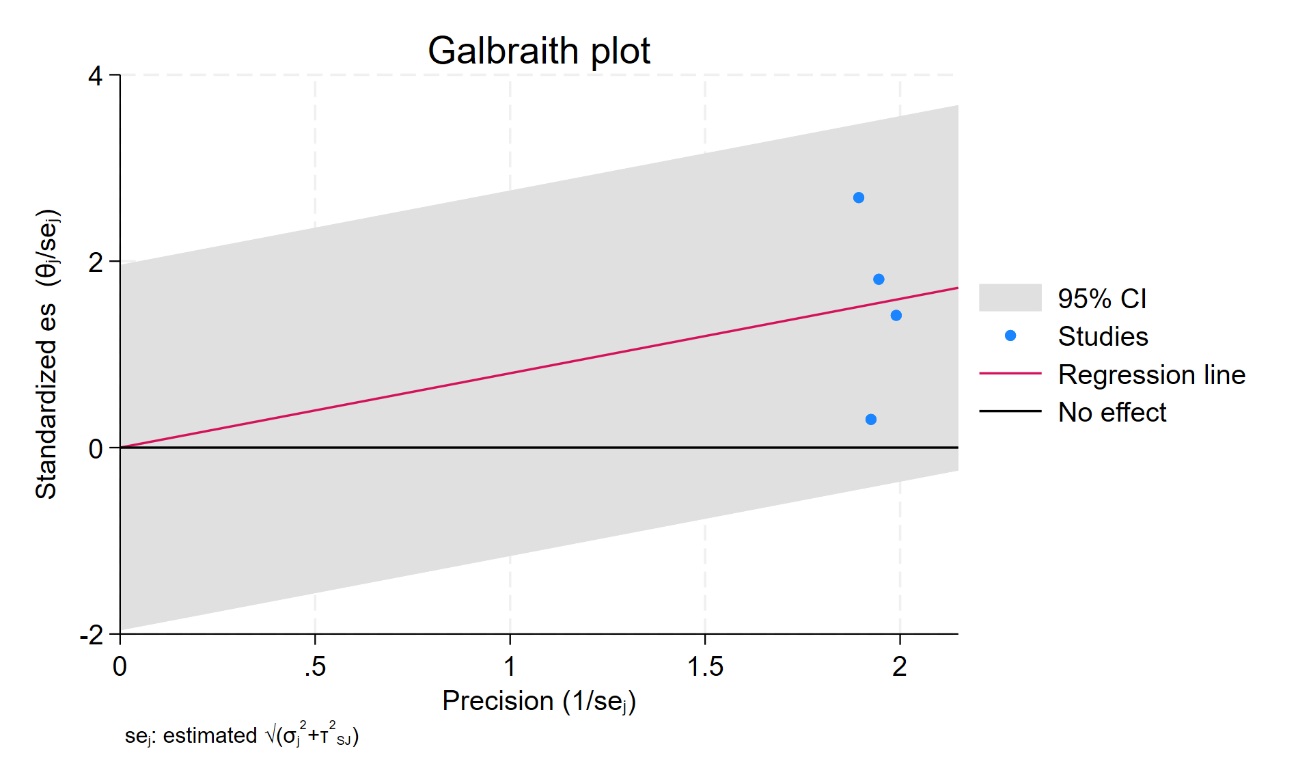


**Figure S8.** Galbraith plots for the risk of cardiovascular mortality.

1. aPWV – Linear.


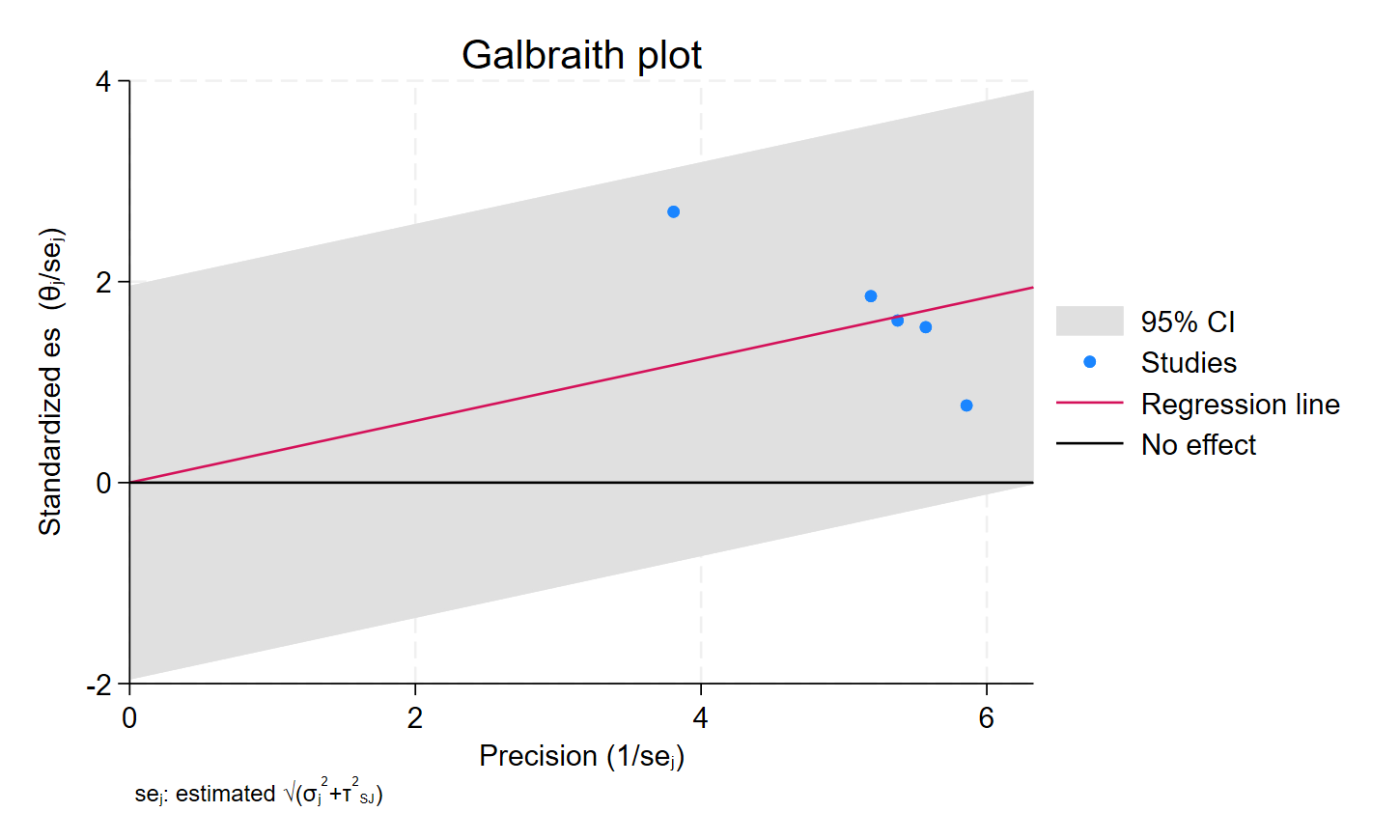


1. baPWV – Categorical.


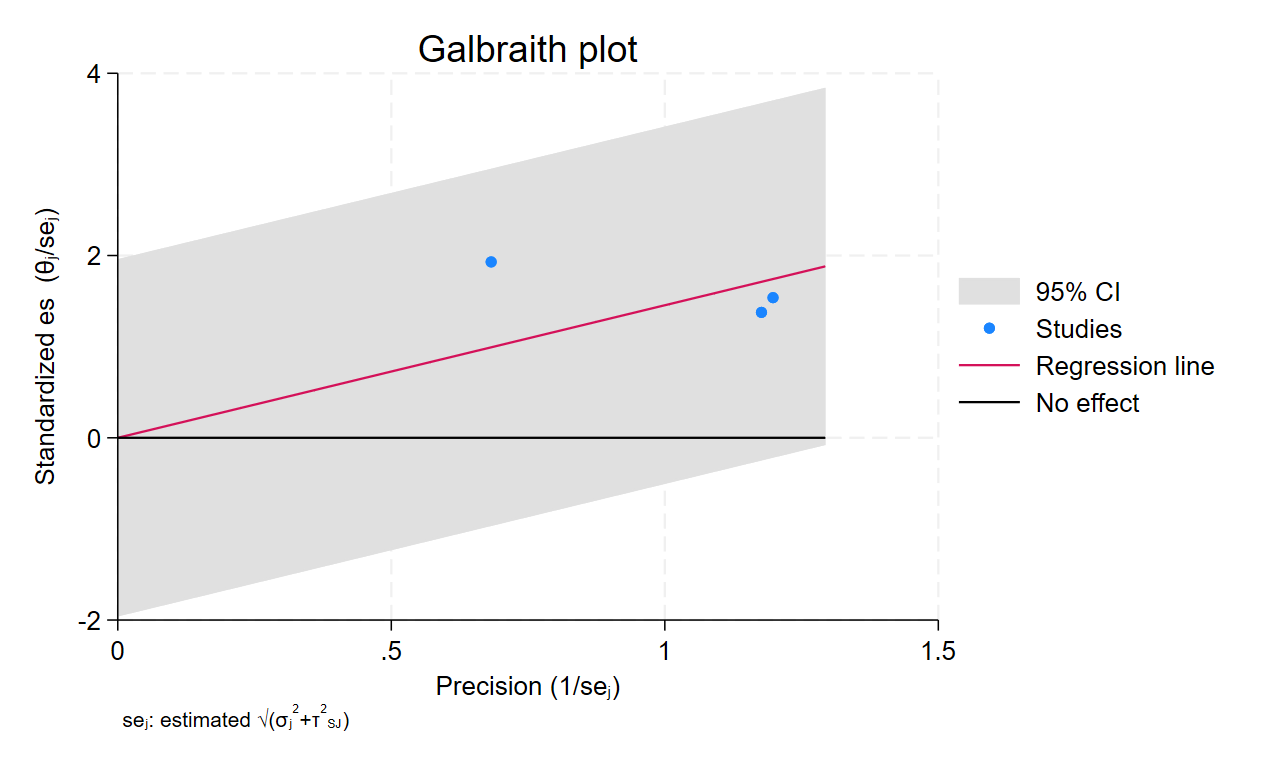


**Figure S9.** Galbraith plots for the risk of cardiovascular disease.

1. aPWV – Linear.


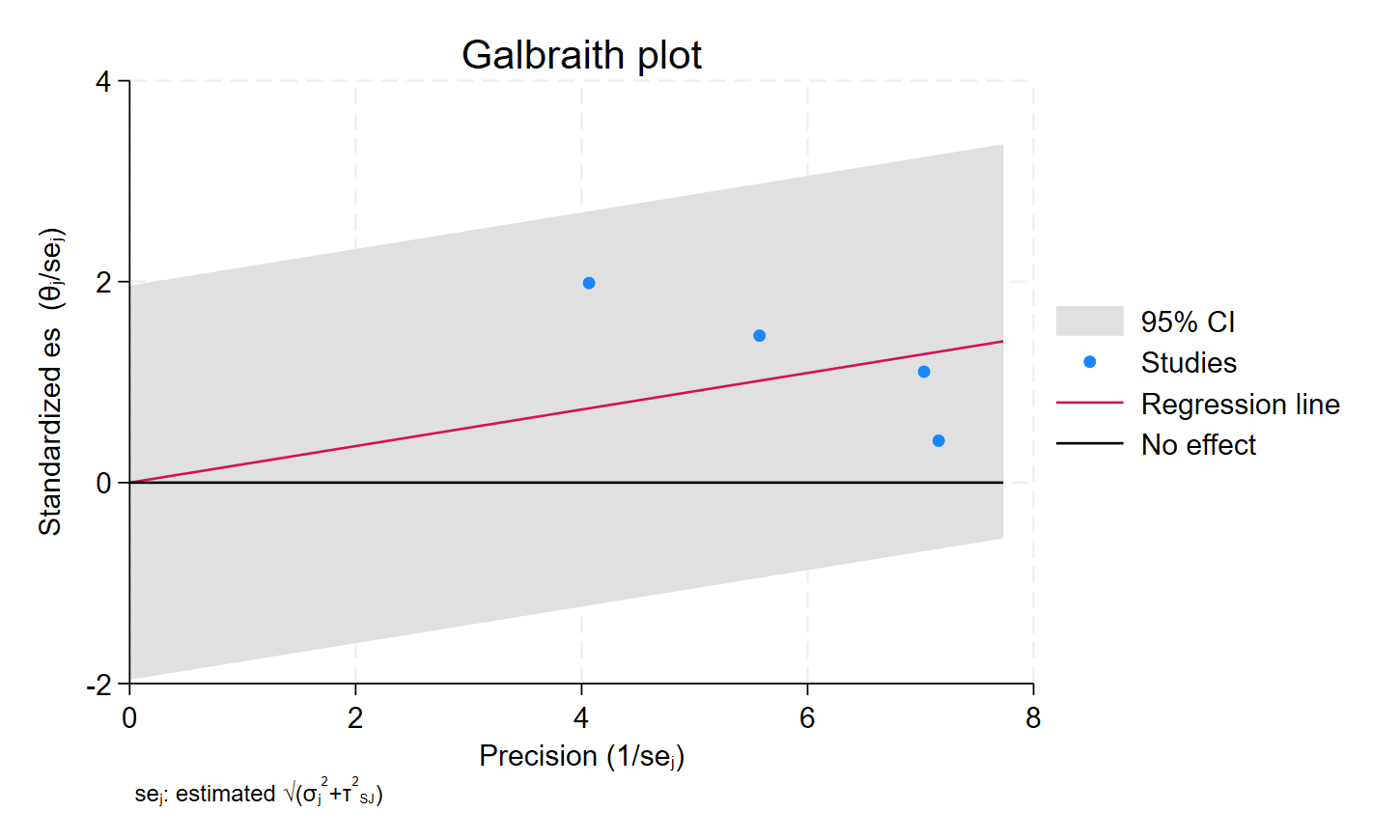


1. baPWV – Categorical.


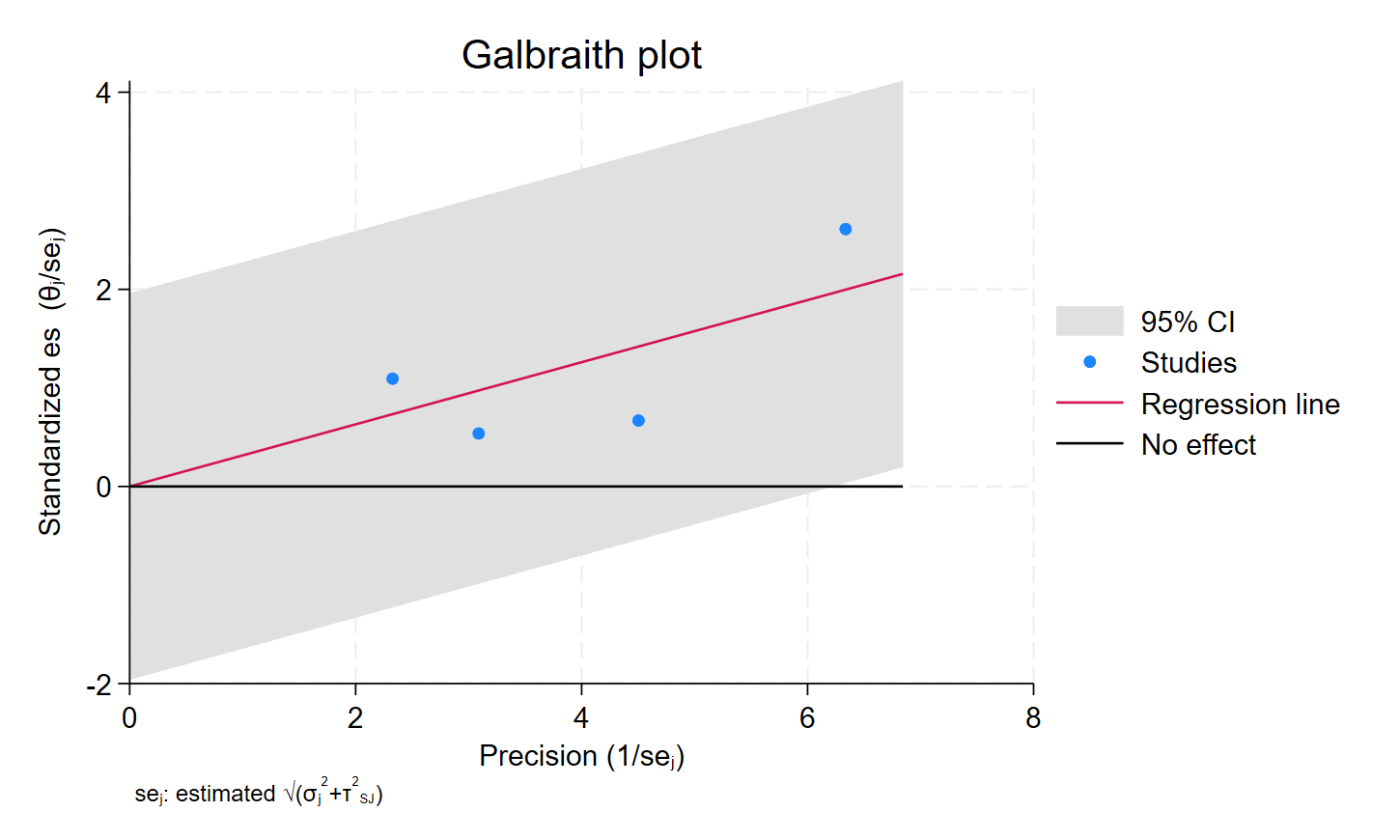


**Appendix S1.** Search strategy.

- Medline (via PubMed), Scopus and Web of Science.

(“arterial stiffness” OR “vascular stiffness” OR “aortic stiffness” OR PWV OR “pulse wave velocity” OR “carotid-femoral pulse wave velocity” OR cfPWV OR “brachial-ankle pulse wave velocity” OR baPWV) AND (kidney disease OR “chronic kidney disease” OR “renal impairment” OR “diabetic kidney disease” OR CKD) AND (mortality OR survival OR cardiovascular OR “major cardiovascular events” OR “cardiovascular events”) AND (hr OR "hazard ratio" OR "odds ratio" OR "risk ratio" OR “relative risk” OR risk)

- Grey literature.

Not specified.
